# Cost-effectiveness of recommended interventions for managing low back pain: A systematic review

**DOI:** 10.1371/journal.pmed.1005273

**Published:** 2026-10-27

**Authors:** Stephanie Mathieson, Martin Underwood, Muhammad Ibrahim, Zhilin Hua, Zhaohua Zhu, Paulo H. Ferreira, Hema Mistry, Emily R. Atkins, Jocelyn L. Bowden, Jillian P. Eyles, Andrew M. Briggs, David J. Hunter, Manuela L. Ferreira

**Affiliations:** 1 Sydney School of Public Health, Faculty of Medicine and Health, The University of Sydney, Camperdown, New South Wales, Australia; 2 Sydney Pharmacy School, Faculty of Medicine and Health, The University of Sydney, Camperdown, New South Wales, Australia; 3 Warwick Medical School, University of Warwick, Coventry, United Kingdom; 4 University Hospitals of Coventry and Warwickshire, Coventry, United Kingdom; 5 The George Institute for Global Health, University of New South Wales, Sydney, New South Wales, Australia; 6 Sydney Musculoskeletal Research Centre, Kolling Institute, The University of Sydney, Sydney, New South Wales, Australia; 7 Charles Perkins Centre Musculoskeletal Hub, School of Health Sciences, Faculty of Medicine and Health, The University of Sydney, Camperdown, New South Wales, Australia; 8 Department of Rheumatology, Royal North Shore Hospital, St Leonards, NSW, Australia; 9 Curtin School of Allied Health, Faculty of Health Sciences, Curtin University, Perth, Western Australia, Australia; Université Paris Cité UFR de Médecine: Universite Paris Cite UFR de Medecine, FRANCE

## Abstract

**Background:**

Low back pain is the leading cause of disability worldwide. Although numerous treatment approaches are recommended by international clinical guidelines, there is still limited understanding of which interventions provide the best value for money. We aimed to critically appraise and summarise the evidence on the cost-effectiveness and costs of interventions endorsed in recent guidelines to manage low back pain (LBP), compared to usual care.

**Methods and findings:**

We searched 10 electronic databases (e.g., MEDLINE (OvidSP), EMBASE (OvidSP), PsycINFO (OvidSP)) from inception to 9th of February 2026 for cost-effectiveness studies of guideline-recommended treatments to manage non-specific LBP. The primary outcome was cost/quality-adjusted life year (QALY), which was converted to 2024 Great British Pound values, tabulated and summarised qualitatively. Secondary outcomes were direct costs (total, interventional, health service use, community services and out-of-pocket costs) and indirect costs (e.g., lost wages, lost productivity, absenteeism). We assessed methodological quality using the Consensus Health Economics Criteria (CHEC) checklist for within-trial economic evaluations or the Philips checklist for modelling studies. Our review included 20 studies (17 within-trial economic evaluations and three modelling studies), published between 2005 and 2026. Half (*n* = 10) focussed on chronic LBP (>3-month duration), and eight included mixed populations (i.e., acute, subacute and chronic). All studies were of non-pharmacological, non-surgical interventions, both single-modality and multimodal interventions (e.g., exercise plus spinal manipulation; diet plus physical activity). Most interventions are cost-effective at a willingness-to-pay threshold of £20,000. Moreover, interventions aiming to reduce fear and stress or support self-management in people with chronic LBP, as well as clinician-delivered interventions including yoga, acupuncture, cognitive therapy and self-management support are potentially cost-saving from the healthcare, societal and payer’s perspectives, as they are both more effective and less costly than usual care (e.g., first contact management, including advice and medication). All of the evidence is derived from high-income countries, and from a considerable heterogenous body of literature. Evidence on cost-effective treatments in low- and middle-income countries remains limited.

**Conclusions:**

Non-pharmacological, non-surgical interventions that include education, psychological support, acupuncture and exercise seem to be cost-effective for managing LBP, compared to usual care. Improved access to and implementation of these effective treatments is urgently needed so more people can benefit from them in routine care.

**Registration:**

The review was registered on PROSPERO (https://www.crd.york.ac.uk; CRD42024510270).

## Author summary

**Why was this study done?:**

- Low back pain is the leading cause of disability worldwide, affecting millions of people and placing a major financial burden on individuals, healthcare systems, and society.
- Many invasive and non-invasive treatments are recommended for low back pain, but understanding which options provide the best value for money is important for healthcare funding and policy decisions.
- Previous reviews have focussed on specific treatments only, whereas a broader assessment of the cost-effectiveness of all available low back pain interventions can better inform decision-makers.

**What did the researchers do and find?:**

- We reviewed all economic evaluations of treatments endorsed by international clinical practice guidelines.
- Several non-drug treatments, such as yoga, acupuncture, physical therapies, and psychological therapies, significantly improve outcomes while also reducing costs compared with usual care.
- Combined treatment approaches—including exercise paired with manual therapy, cognitive behavioural therapy, or dietary interventions—were good value treatments for people with persistent low back pain.

**What do these findings mean?:**

- These findings suggest that investing in effective non-surgical and multidisciplinary care can improve health outcomes while making efficient use of healthcare resources**.**
- The priority now is to increase access to and implementation of these effective treatments so more people can benefit from them in routine care.
- Most evidence comes from high-income countries, and therefore the relevance of our findings for populations of low- and middle-income settings is unknown.

## Abstract 3

In a systematic review of studies on low back pain interventions, Stephanie Mathieson and colleagues assess the cost-effectiveness of guideline-endorsed recommendations of non-surgical and non-pharmacological interventions.

## Background

Low back pain (LBP) is the leading cause of years lived with disability globally [1]. The economic impact of LBP extends beyond the direct healthcare system costs to affect individuals, employers, health and social care systems and society. The average annual direct and indirect costs (e.g., work absenteeism) are substantial [2]. For instance, in Australia, ~$3.4 billion (~Great British Pound [GBP]1.7 billion) was spent on direct health costs for managing back problems in 2020/21, equating to 2.2% of Australia’s total health system expenditure [3]. A 2023 meta-analysis of the global economic burden of LBP determined that the pooled annual direct and total (i.e., direct plus indirect) costs of LBP per patient were United States Dollar (USD) 9,231 (GBP 6,994) and USD 10,143 (GBP 7,685), respectively [2].

Many interventions that can be delivered by a range of health workers are available to manage LBP. Most clinical guidelines recommend providing advice and educational strategies for people experiencing LBP, including re-engagement in physical activity and exercise, and supporting self-management of LBP symptoms to improve function [4]. While other interventions may be needed to manage symptoms in people with acute or chronic LBP, such as medicines, their judicious use should not be the only focus of care. More invasive interventions such as spinal injections may be considered when non-invasive treatments have not provided a sufficient benefit, and a clear clinical indication supports their use. With many different options available, information on the cost-effectiveness of interventions to manage LBP is critical to those making funding decisions. A 2011 systematic review (26 studies) investigated the cost-effectiveness of interventions recommended in the 2007 American College of Physicians and American Pain Society joint LBP clinical guidelines [5, 6]. The authors concluded that the evidence supported the cost-effectiveness of interdisciplinary rehabilitation, exercise, acupuncture, spinal manipulation and cognitive behavioural therapy. The studies included in the review showed substantial heterogeneity in their comparison interventions, limiting the transferability of their findings to policy and practice. In 2018, Miyamoto, in a systematic review (16 studies) of the cost-effectiveness of exercise for LBP, concluded that it may be cost-effective when compared to usual care (e.g., first contact management) [7]. In 2018, Salathé, in a systematic review of the effectiveness of multidisciplinary biopsychosocial rehabilitation (MBR; 13 studies), found some evidence to support its cost-effectiveness [8]. In 2017, Andronis, in a systematic review (33 studies), found that combined physical and psychological treatments, information and education interventions, and manual therapies could be cost-effective, but that there was inconsistent evidence on the cost-effectiveness of physical exercise programmes [9]. Since these reviews were published, there have been new recommendations from high-quality guidelines for the management of LBP, informed by new evidence from robust clinical trials [10–12]. No review has evaluated the cost-effectiveness of all interventions recommended in contemporary clinical practice guidelines, compared to usual care. Summarising and evaluating the most updated evidence of the cost-effectiveness of interventions recommended for LBP care in clinical practice guidelines can support policymakers and health services in decisions about funding of interventions and their availability. This is particularly relevant in the context of determining essential care packages for Universal Health Coverage. We have conducted a systematic review to critically appraise and summarise the evidence on the cost-effectiveness and costs of interventions recommended (in favour) in recent guidelines to manage LBP, compared to usual care.

## Methods

The protocol for this systematic review followed the Preferred Reporting Items for Systematic Reviews and Meta-Analyses (PRISMA-P and PRISMA) and Consolidated Health Economics Evaluation Reporting Standards (CHEERS) statement. The review was registered on the International Prospective Register of Systematic Reviews (PROSPERO) (https://www.crd.york.ac.uk; CRD42024510270). This study is reported as per the Preferred Reporting Items for Systematic reviews and Meta-Analyses (PRISMA) statement (S1 Checklist).

### Patient and public involvement

Consumer representatives were not involved in the design not the conduct of this systematic review. Consumer representatives and those with lived experience will be involved in the dissemination of the study findings, according to the Consumer Engagement and Involvement Framework of The George Institute for Global Health, Australia.

### Search strategy

We searched electronic databases from inception to the 9th February 2026 using a search strategy developed following published Cochrane reviews on back pain and economic analyses and librarian input. We searched MEDLINE (OvidSP), EMBASE (OvidSP), PsycINFO (OvidSP), Cochrane Central Register of Controlled Trials (CENTRAL) (OvidSP), CINAHL Complete (EBSCOhost), EconLit with full text (EBSCO), The Tufts Cost-effectiveness Analysis Registry (CEA), NHS Economic Evaluation Database (OvidSP), Health Technology Assessment (HTA) (OvidSP), and International Pharmaceutical Abstracts (OvidSP) with no restrictions. The search strategy is detailed in S1 File and was adapted for each database. We also conducted a manual search of relevant systematic reviews and email communication with content experts to identify missing literature.

### Eligibility

#### Types of studies.

We included cost-effectiveness evaluations based on randomised controlled trials or modelling studies of eligible interventions for the management of LBP. We considered model-based economic evaluations that relied on inputs from randomised trials (i.e., micro-simulation modelling) or external information that provides inputs from multiple cost sources. We excluded Phase I and II clinical trials, opinion pieces, editorials, abstracts, case series reports and reviews.

#### Population.

We included studies with adult (≥18 years) participants with non-specific LBP (where the experience of pain is not reliably attributed to nociceptive input from a specific structure) with or without leg pain. We excluded studies on participants with radiculopathy (or sciatica) only, or where the primary area of pain was not the lower back (i.e., between the 12th rib and the gluteal folds). We also excluded studies that enrolled participants with LBP attributed to distinct pathologies, such as cancer, osteoporosis, infection, fracture, trauma, and autoimmune inflammatory diseases, as well as studies focussing on participants that had undergone spinal surgery within the last 12 months, those who were pregnant or did not present data separately for LBP if mixed populations were sampled (e.g., lumbar and cervical pain). We considered the experience of LBP as acute (less than 6 weeks), subacute (6–12 weeks) or chronic (greater than 12 weeks).

#### Intervention.

We included studies that assessed LBP interventions as recommended by at least one of the following guidelines: World Health Organization [12], National Institute for Health and Care Excellence [10], and North American Spine Society [11]. We only included studies assessing interventions where the direction of the recommendation was in favour of using/offering the intervention in at least one of these guidelines, regardless of the strength of the recommendation (e.g., strong or conditional), certainty of the evidence, or dose/duration (S2 File). We did not include guidelines that were greater than five years old at the time of protocol development [13], focussed on one class of interventions to manage LBP (e.g., physical therapies alone) [14], or on one discrete population (e.g., veterans). We did not include clinical care standards designed for quality improvement in key areas (e.g.[15],). We did not include studies that reported the use of health services, such as studies of claim databases where no intervention was implemented.

#### Comparator.

In the current report, we have only included studies using sham/placebo, usual care (i.e., first contact care), or no treatment controls. This was decided as the focus of the current review is on the cost-utility of guideline-recommended interventions that may be commissioned, rather than the comparative cost-effectiveness of these interventions.

### Outcomes

Our primary outcome was the incremental cost per quality-adjusted life year (QALY) gained by the intervention group compared with the comparator, as reported by studies of relevant, included interventions. We also report willingness to pay (WTP) according to the thresholds as reported in the original studies, and any incremental net monetary benefit analyses. Secondary outcomes included direct costs (total costs, intervention costs, costs with health service use, community services costs, and out-of-pocket costs) and indirect costs (e.g., lost wages, lost productivity, and absenteeism). To evaluate the specific economic burden on workers, we defined direct costs as the immediate healthcare resources consumed by the intervention, while indirect costs were the broader societal productivity losses, explicitly capturing work absenteeism, presenteeism, and lost wages as extracted from the primary trial data.

### Screening

Two review authors from a panel (SM, MI, ZH, MU, ZZ, MLF) independently screened and identified titles and abstracts to determine eligibility. We excluded duplicate studies. Two reviewers independently appraised the full texts of potentially eligible studies to determine inclusion. Disagreements were resolved by discussion first. If needed a third independent reviewer (MLF, MU) arbitrated. For articles written in languages that the authors could not read, the authors sought colleagues to assist with reading the article or used Google Translate to screen for eligibility.

### Data extraction and management

Two review authors from a panel (SM, MU, MI, ZH, ZZ, PHF, MLF) independently extracted data using standardised, piloted data extraction forms in Covidence. Disagreements were resolved by discussion first. If needed, a third independent reviewer (MLF, MU) arbitrated. The reviewers did not assess any of their own studies. We extracted the following information when available on bibliometric data (authors, title, year of publication, language, country, funding sources); study characteristics (setting, sample size, study design, e.g., randomised trial, modelling study, sampling details); participants (age, sex, race/ethnicity, diagnosis/LBP classification, symptom duration); interventions and controls (type, dose, duration, mode of delivery); outcome data (type of economic evaluation, incremental cost-effectiveness measures of base-case analyses, type of costs evaluated and details for secondary outcomes, perspective (e.g., societal, health system, etc.), time horizon, currency, price year, discount rates, and tariffs).

### Quality assessment

Two reviewers from a panel (SM, MLF, ZZ, HM) independently assessed the methodological quality of eligible studies using the 19-item Consensus on Health Economic Criteria (CHEC) checklist for randomised trials [16], and the Philips checklist for modelling studies (S3 File) [17]. Disagreements were arbitrated by a third reviewer (PHF) if consensus was not reached.

### Strategy for data synthesis and analysis

The flow of studies is summarised in a study flow diagram, following the PRISMA statement [18]. We reported study and participant characteristics descriptively and dichotomous variables as proportions, *n*/*N* (%). We reported continuous variables using point and variability estimates (e.g., means and standard deviations or medians and interquartile ranges). We tabulated and qualitatively summarised the primary outcomes (cost per QALY) of cost-effectiveness evaluations. Where available, we also presented the average cost difference between interventions, QALY (and 95% confidence intervals [CI]), and WTP for one QALY. We presented costs in the original study currency as well as in 2024 GBP (Great British Pounds (£)). We first inflated cost estimates reported in each study to 2024 price levels using country-specific Consumer Price Index (CPI) data obtained from the Organisation for Economic Co-operation and Development (https://www.oecd.org/en.html), relative to the original study year. We then converted costs expressed in 2024 local currency to 2024 British pounds (£) by dividing by the corresponding 2024 country-specific Purchasing Power Parity (PPP) for gross domestic product (GDP). We set our notional WTP threshold at £20,000 Great British Pounds per QALY, as it corresponds to the current NICE recommended threshold. This was done to maintain contextual precision across different international healthcare systems. We standardised all cost and utility values by extracting the primary data, adjusting for inflation using the localised CPI relative to the specific year of each study’s execution, and converting them into a baseline currency to ensure accurate and meaningful cross-contextual comparisons.

We present the results separately for different perspectives: healthcare, societal, payer or employer. We classified the income status of countries using the 2025 World Bank income classification into low-income, middle-income (upper- or lower-middle), or high-income [19]. The heterogeneity of study populations, interventions, perspectives, and the health and social care systems in which the work was done made any data pooling inappropriate. Moreover, we deemed pooling inappropriate due to the absence of measures of variance for economic outcomes. We assessed between-study heterogeneity qualitatively across structural, clinical, and methodological domains prior to synthesis, including (1) disparate economic perspectives; (2) highly variable time horizons (≤12 months to lifetime models); and (3) heterogeneous geographic cost structures. We present a narrative synthesis to preserve the contextual validity of the findings.

Likewise, while the study protocol initially planned for a Grading of Recommendations Assessment, Development and Evaluation (GRADE) assessment of the primary outcomes, during data synthesis we determined that the currently available GRADE frameworks [20] would be methodologically unsuited for evaluating synthesised economic evidence and therefore not included in this review. Generative AI was not used in this study.

## Results

The initial search retrieved 9,285 records, of which 20 papers reporting on 24 studies and providing the cost-effectiveness of 55 comparisons with usual care (e.g., general practitioner-delivered first contact care, usual physiotherapy care) were included [21–40]. Fig 1 presents a flow of studies. Studies were published between 2005 [34] and 2026 [40] and were from 9 high-income countries, most frequently from the UK (*n* = 7 studies, 37%, Table 1). Most studies (*n* = 17) were cost-effectiveness analyses of randomised trial data. There were 3 model-based studies [32, 35, 36]. We identified one study [35] that used original data from 17 trials to estimate cost-utility for different interventions against usual care controls for a standardised back pain population. Of these studies, 10 met our inclusion criteria, four of which [21, 24, 29, 34] had already been identified and reported in our review. The remaining six provided new data on 10 comparisons and were included in our economic analysis [41–46].

**Table 1:** Study and participant characteristics of included studies.

Study	Study design	Country	Total number of participants	Symptom duration	Mean age (Years)	Age SD (Years)	Female (*n*)	Male (*n*)
Chuang (2012)	RCT	UK	313	Chronic	46.3	11.4	220	93
Darlow (2019)	Cluster RCT	New Zealand	226	Acute, subacute and chronic	46.1	14.5	105	121
Geraghty (2024)	RCT	UK	825	Acute, subacute and chronic	54.0	15	479	342
Herman (2017)	RCT	USA	342	Chronic	49.6	12.1	200	142
Herman (2020)	Modelling	USA						
Cherkin (2001)*	RCT	USA	262	Chronic	44.9	11.5	152	110
Cherkin (2009)*	RCT	USA	638	Chronic	47	13	396	242
Cherkin (2011)*	RCT	USA	401	Chronic	48	11	258	143
Haake (2007)*	RCT	German	1,162	Chronic	49.6	14.6	692	470
Hurwitz (2002)*	RCT	USA	681	Subacute and chronic	51	16.7	354	327
VonKorff (2005)*	RCT	USA	240	Chronic	49.8	9.8	150	90
Hochheim (2021)	RCT	Germany	223	Chronic	55.2	7.9	79	144
Herman (2026)	RCT	USA	672	Chronic	74.1	5.9	406	266
Hollinghurst (2008)	RCT	UK	291^†^	Chronic	45.5	10.5	NR	NR
Johnson (2007)	RCT	UK	234	Chronic	47.3	10.9	140	94
Kent (2023)	RCT	Australia	329^#^	Chronic	47.3	15.3	197	132
Kim (2010)	Modelling	South Korea	2,982	Chronic	NR	NR	NR	NR
Kongstad (2024)	RCT	Denmark	297	Acute, subacute and chronic	50.7	14.5	164	133
Lamb (2010)	RCT	UK	701	Subacute and chronic	54.0	14.9	420	281
Lambeek (2010)	RCT	Netherlands	134	Chronic	46.2	9.1	56	78
Ratcliffe (2006)	RCT	UK	241	Acute, subacute and Chronic	42.0	10.8	146	95
Skonnord (2022)	RCT	Norway	167	Acute	33.9	11.4	85	82
Suman (2019)	Cluster RCT	Netherlands	779	Acute, subacute and chronic	56.2	14.3	440	339
UK BEAM (2005)	RCT	UK	1,287	Subacute, chronic	43.1	11.2	721	566
Verhaeghe (2018)	Modelling	Belgium	1,298	NR	NR	NR	NR	NR
Williams (2019)	RCT	Australia	159	Chronic	56.7	13.5	94	65

*Studies included in Herman 2020.

† Total number of participants allocated to usual care or massage.

# Total number of participants allocated to cognitive functional therapy only or usual care.

Abbreviations: RCT, randomised controlled trial; *n*, number; UK, United Kindom; USA, United States of American; NR, not reported.

**Fig 1:**
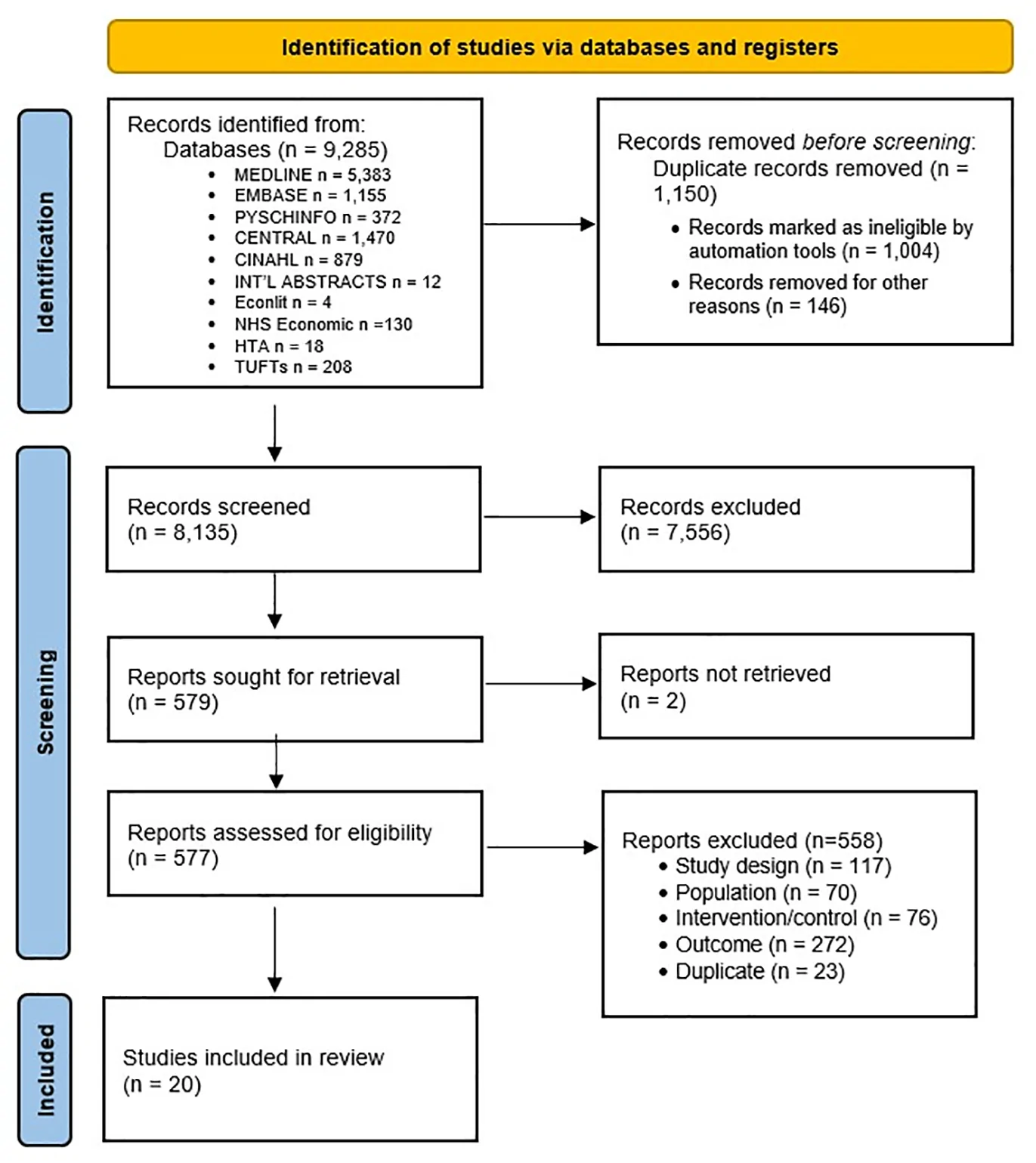
PRISMA flow diagram of included studies.

Total number of participants included in the cost-effectiveness analyses was 14,884 with a mean sample size per comparison of 266 [range: 134 [30] to 2,982 [32] (Table 1)]. Participants’ mean age ranged from 45.5 [26] to 74.1 years [40]. Sex or gender was reported in 16 studies. Among those reporting this information, 5,954/10,309 female participants (58%) were included in the cost-effectiveness analyses. Approximately half of the studies were of people only experiencing chronic symptoms (*n* = 10 studies; 55%), with eight of the remaining studies including a population of mixed LBP duration (Table 1). One study only included participants with acute symptoms [39], while the remaining study did not report the symptom duration [32]. We detail the characteristics of the included studies and their participants in Table 1.

### Intervention characteristics

All studies were of non-pharmacological, non-surgical interventions, with most assessing the cost-effectiveness of acupuncture (5 studies, 16 comparisons) [35–37, 39, 40], or cognitive and psychological therapies (6 studies, 7 comparisons) [23, 24, 27, 29, 30, 38]. Included studies also evaluated the cost-effectiveness of single-modality interventions such as exercises [34], Yoga [21], massage [26, 41, 45], spinal manipulation [32, 34], and multimodal interventions, including multidisciplinary care [25, 43], combined exercise and spinal manipulation [34], exercise and cognitive therapy [38], education and advice [22, 28, 31] or lifestyle-based intervention [33], compared to usual care. In the included studies, usual care was broadly defined as first-contact care and mostly comprised of education (e.g., Back School booklet), limited pharmacotherapy, advice to stay active and when needed, or referral to a physiotherapist (Table 2). Suman and colleagues [31] provided participants in the usual care, a digital patient information letter, and Williams and colleagues [33] defined usual care as normal care delivered while on the waitlist to receive surgery. Fig 2 provides a summary of the types, frequency and guideline endorsement of included interventions.

**Table 2:** Description of included interventions.

Study	Treatments investigated	Comparator	Study conclusions
Cherkin (2001)*	Therapeutic massage; Traditional Chinese Medical acupuncture	Usual care including self-care education	See Herman (2020)
Cherkin (2009)*	Standardised acupuncture; Individualised acupuncture	Usual care including analgesics, general practitioner care and occasionally physiotherapy	See Herman (2020)
Cherkin (2011)*	Relaxation massage; Spinal manipulation	Usual routine medical care (unspecified)	See Herman (2020)
Chuang (2012)	Yoga	Usual care from a general practitioner	In the basis of this trial, 12 weekly group classes of specialised yoga are likely to be a cost-effective intervention for treating patients with chronic or recurrent LBP.
Darlow (2019)	Fear Reduction Exercised Early (FREE) method [restructures LBP consultations to prioritise early identification and management of barriers to recovery]	Usual care from a general practitioner	Findings from this study suggest that the FREE approach improves GP concordance with LBP guideline recommendations but does not improve patient recovery outcomes compared with usual care. The FREE approach may reduce unnecessary healthcare use and produce economic benefits.
Geraghty (2024)	Usual care + SupportBack internet behavioural support or usual care + SupportBack + physiotherapist telephone support	Usual general practitioner care	SupportBack group dominated usual care (more effective and less costly). Both SupportBack interventions likely (40%–60%) to be cost-effective at a threshold of £20,000 per quality adjusted life year compared with usual care.
Haake (2007)*	Verum acupuncture	Usual care including medication, advice and occasionally physiotherapy	See Herman (2020)
Herman (2017)	Cognitive Behavioral Therapy (CBT) or Mindfulness-Based Stress Reduction (MBSR)	Usual care including traditional medical care participants were already receiving	In this setting, CBT and MBSR have high probabilities of being cost-effective, and MBSR may be cost-saving, as compared to usual care for adults with chronic LBP.
Herman (2020)	Used original data from 17 trials of which 6 were included in our report.	Usual medical and self-care	Markov modelling facilitates comparisons across interventions not directly compared in trials, using consistent outcome measures after balancing the baseline mix of patients. Outcomes differed by pain impact level, emphasising the need to measure chronic LBP subgroups
Herman (2026)	Standard acupuncture (SA, > 15 sessions in 12 weeks) or Enhanced acupuncture (EA, additional 6 sessions in 12 weeks)	Usual medical care	EA dominated usual care from both the healthcare and payer’s perspectives. The incremental cost-effectiveness ratio for SA compared to usual care was $52,897/QALY from the healthcare perspective and $58,528/QALY from the payer’s perspective. Both likely to be cost-effective at a probability of 99.6% (EA) and 74.8% (SA), and threshold of $100,000/QALY.
Hochheim (2021)	Multidisciplinary biopsychosocial rehabilitation (MBR)	Usual recommended primary care including advice, and exercises	This first cost-effectiveness study with combined data from a private health insurer and a controlled trial in Germany demonstrated that long-term MBR for the treatment of chronic LBP is cost-effective. Subgroups with major impairment from back pain benefitted more from the intervention than those with minor impairment. MBR significantly reduced sick leave in all participants.
Hollinghurst (2008)	Massage or Alexander technique^#^	Usual care (home-based exercise and advice)	Massage alone was not cost-effective when compared to usual care, with an average cost of £204 and no gain in QALY.
Hurtwitz (2002)*	Physical therapy	Usual care including advice on self-management and analgesics	See Herman (2020)
Johnson (2007)	Exercise + CBT	Usual medical care and educational booklet	This intervention program produces only modest effects in reducing LBP and disability over a 1-year period. The observation that patient preference for treatment influences outcome warrants further investigation.
Kent (2023)	Cognitive Functional Therapy (CFT) with or without movement sensor biofeedback^†^	Usual care pathway including physiotherapy, massage, medication or chiropractic care	CFT can produce large and sustained improvements for people with chronic disabling LBP at considerably lower societal cost than that of usual care.
Kim (2010)	Acupuncture + usual care	Usual routine medical care (unspecified)	Acupuncture collaborative therapy for patients with chronic LBP may be cost-effective if the usual threshold is applied. Further empirical studies are required to overcome the limitations of uncertainties and improve the precision of the results.
Kongstad (2024)	Telephone application-based educational support on self-management + usual care	Usual care according to the advice or treatment offered by their healthcare professional	From healthcare perspective, the app self-management support intervention is very likely (98%) to be cost-effective at a threshold of £20,000. Including productivity loss to results, introduces uncertainty.
Lamb (2010)	CBT	Usual care from a general practitioner	A bespoke cognitive behavioural intervention package for LBP has an important and sustained effect at 1 year on disability from LBP at a low cost to the healthcare provider.
Lambeek (2010)	Workplace intervention [graded activity, ergonomics to restore occupational functioning]	Usual care from a medical specialist, general practitioner, or allied health profession- als	Implementation of an integrated care programme for patients sick listed with chronic LBP has a large potential to significantly reduce societal costs, increase effectiveness of care, improve quality of life, and improve function on a broad scale. Integrated care therefore has large gains for patients and society as well as for employers.
Ratcliffe (2006)	Traditional Chinese Acupuncture	Usual care from a general practitioner	Acupuncture care for LBP seems to be cost-effective in the longer term.
Skonnord (2022)	Acupuncture	Usual care including advice, medication and any eventual sick leave	Acupuncture may be cost-effective from a 1-year perspective, but more studies are needed.
Suman (2019)	Multifaceted eHealth strategy [to reduce negative beliefs and improve their knowledge and self-management]	Usual medical care including digital patient information	A multifaceted eHealth strategy was not effective in improving patients’ back pain beliefs or in decreasing disability and absenteeism but showed promising cost-utility results based on QALYs.
UK BEAM	Exercise programme or spinal manipulative therapy or both	Best care in general practice	Spinal manipulation is cost-effective when added to usual care and compared to usual care in general practice. Spinal manipulation alone gives better value for money than when combined with exercises.
Verhaeghe (2018)	Osteopathy	Usual care including general practitioner consult, pain medication, physiotherapy, rehabilitation, medical imaging, or hospitalisation	Osteopathy was found to be a ‘dominant’ and cost-effective strategy (neck pain) compared to usual care.
Von Korff (2005)*	Multidisciplinary intervention	Usual care including analgesics, general practitioner care, and occasionally physiotherapy	See Herman (2020)
Williams (2019)	Healthy lifestyle intervention [weight loss, physical activity and diet]	Usual care received while on the waitlist for orthopaedic consultation	The healthy lifestyle intervention seems to be cost-effective from the societal perspective. However, variability in the sensitivity analyses indicates caution is needed when interpreting these findings.

*Studies included in Herman (2020).

† CFT with biofeedback is not endorsed by clinical practice guidelines and was not included in our main results.

# The Alexander technique is not endorsed by clinical practice guidelines and was not included in our main results.

Abbreviations: LBP, Low Back Pain; FREE, Fear Reduction Exercised Early; GP, general practice; CBT, Cognitive Behavioural Therapy; MBSR, Mindfulness-Based Stress Reduction; SA, Standard Acupuncture; EA, Enhanced Acupuncture; QALY, Quality-Adjusted Life Year; MBR, Multidisciplinary Biopsychosocial Rehabilitation; CFT, Cognitive Functional Therapy.

**Fig 2:**
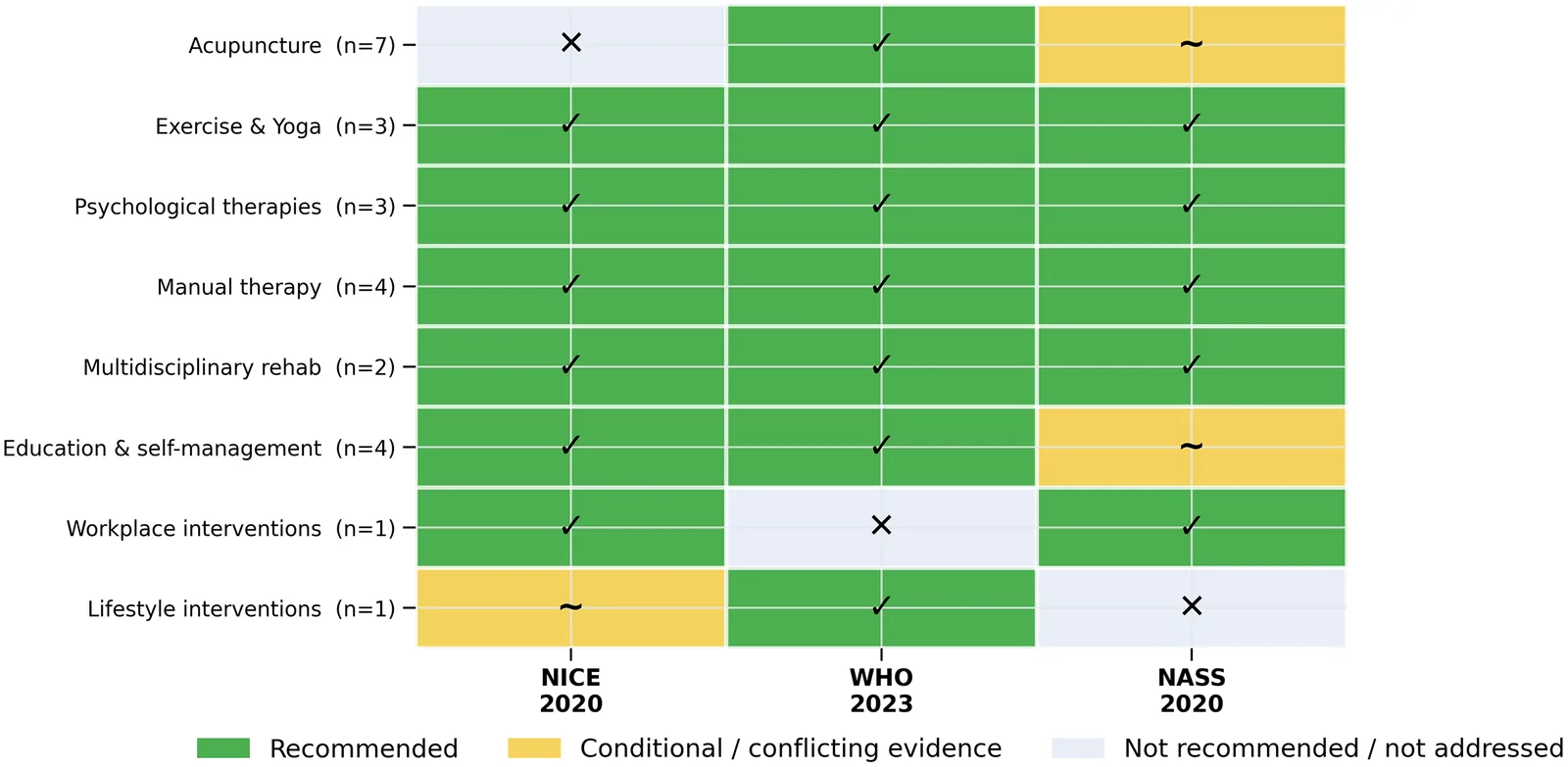
Interventions Evaluated and Alignment with the National Institute for Health and Care Excellence (NICE), World Health Organization (WHO) and North American Spine Society (NASS) Clinical Practice Guidelines.

### Economic evaluation characteristics

All studies were from high-income countries. Eighteen comparisons from 13 studies [21–24, 26, 28–30, 33, 34, 38–40] considered a healthcare system perspective, 21 comparisons (10 included studies [21, 22, 24, 27, 28, 30, 31, 33, 35, 37]) considered a societal perspective, and 14 comparisons (4 studies [22, 25, 35, 40]) a payer perspective. The probability of being cost-effective at a WTP threshold was reported in 21 comparisons and ranged from 50.0% to 99.6% probability of cost-effectiveness over usual care (Table 3).

**Table 3:** Main results of cost-effectiveness analyses of interventions compared to usual care.

Study	Intervention	Country	Time horizon Months	Cost of intervention (95% CI)	QALY (95% CI)	Cost/ QALY (GBD values)	Willingness to pay (WTP) for one QALY
**Health care perspective**							
Darlow (2019) [22] *N*=126	GP training in fear reduction	New Zealand	6	−NZD270 (−732, 192) (−£159 (−431, 113))	0.005 (−0.014, 0.024)	Intervention dominant	~80% at WTP NZD55,615 (£32,740)
Geraghty (2024) [23] *N*=275	SupportBack self-management	UK	12	−£16 (−128 to 95) (−£19 (−156, 116))	0.011 (−0.0014, 0.039)	Intervention dominant	~60% at WTP < £20,000
Herman [24] 2017 *N*=101	Mindfulness-Based Stress Reduction	USA	12	−USD127 (−2,670, 942) (−£128 (−2,690, 949))	0.034 (0.008, 0.060)	Intervention dominant	90% at WTP < USD50,000 (£50,374)
Herman (2026) [40] *N*=269	Enhanced Acupuncture	USA	12	−USD491 (−2,861, 1,144) (−£396 (−2,308, 923))	0.037 (0.013, 0.062)	Intervention dominant	~90% USD20,000 (£14,697)
Skonnord (2022) [39] *N*=81	Acupuncture	Norway	12	−USD20 (−£16)	0.0350	USD572 (£459)	~95.9% at NOK 275,000 (£24,704)
Lamb (2010) [29] *N*=468	CBT	UK	12	£178 (£245)	0.099	£1,786 (£2,464)	90% at ~ £3,000 (£ 4,141)
Kongstad (2024) [28] *N*=148	App based self-management support	Denmark	9	€230 (−136, 595) (£180 (−106, 465))	0.03 (0.01, 0.05)	€7,336 (£5,737)	98% at ~€23,250 (£18,181)
UK BEAM (2004) [34] *N*=333	SMT+ Exercise	UK	12	£125 (21, 228)^*^ (£214 (36, 391))	0.033 (−0.001, 0.067) *	£3,800 (£6,512)	N/A
Geraghty (2024) [23] *N*=276	SupportBack self-management + telephone support	UK	12	£96 (−14, 206) (£117 (−17, 251))	0.013 (−0.011, 0.037)	£7,336 (£8,935)	30%-40% at WTP £20 000
UK BEAM (2004) [34] *N*=353	SMT	UK	12	£195 (85, 308)^*^ (£334 (146, 528))	0.041 (0.016, 0.066) *	£4,800 (£8,225)	73% at £10,000 (£18,850)
Johnson (2007) [38] *N*=78	Exercise + CBT	UK	12	£27 (−159, 213) (£45 (−263, 352))	NA	£5,000 (£8,265)	90% at £30,000 (£49,590)
UK BEAM (2004) [34] *N*=310	Exercise	UK	12	£140 (3, 278) (£240 (5, 476))	0.017 (−0.017, 0051)	£8,300 (£14,223)	NA
Williams (2019) [33] *N*=80	Healthy lifestyle intervention	Australia	6	AUD386 (−188, 688) (£230 (−112, 410))	0.02 (0.00, 0.005)	AUD19,036 (£11,189)	90% at AUD98,000 (£58,406)
Chuang (2012) [21] *N*=313	Yoga	UK	12	£507 (159, 855) (£715 (224, 1,206))	0.037 (0.006, 0.069)	£13,606 (£19,327)	72% at £20,000 (£28,220)
Herman (2026) [40] *N*=265	Standard Acupuncture	USA	12	USD759 (−755, 2,439) (£612 (−609, 1,968))	0.014 (−0.014, 0.043)	USD52,897 (£42,673)	~27% at US $20,000 (£16,130)
Herman (2017) [24] *N*=98	CBT	USA	12	USD984 (−1,075, 3,385) (£991 (−1,083, 3,410)	0.041 (0.015, 0.067)	USD24,000 (£24,180)	81% at WTP < USD50,000 (£50,374)
Hollinghurst (2008) [26] *N*=147	Massage	UK	12	£204 (151, 257) (£324 (240, 408))	−0.01 (−0.05, 0.04)	Control Dominant	N/A
**Societal Perspective**							
Chuang (2012) [21] *N*=313	Yoga	UK	12	−£213 (−1,028, 600) (−£293 (−1,412, 824))	0.037 (0.006, 0.069)	Intervention dominant	89% at £10,000 (£13,740) and 95% at £20,000 (£27,480)
Darlow (2019) [22] *N*=126	GP training in fear reduction	New Zealand	6	−NZD337 (−1,842, 1,168) −£198 (−1,085, 687)	0.005 (−0.014, 0.024)	Intervention dominant	~75% at WTP NZD55,615 (£32,740)
Kent (2023) [27] *N*=164	Cognitive functional therapy	Australia	12	−AUD5,276 (−10,529, −24) (−£2,671 (−5,331, −12))	0.12 (0.08, 0.16)	Intervention dominant	NA
Lambeek (2010) [30] *N*=66	Workplace advice and graded activity	The Netherlands	12	−£5,310 (−10,042, −391) (−£7,315 (−13,832, −538))	0.09 (0.01, 0.16)	Intervention dominant	NA
Suman (2019) [31] *N*=331	Multifaceted eHealth self-management	The Netherlands	12	−€535 (−2,230, 1,172) (−£587 (−2,445, 1,285))	0.03 (0.001, 0.042)	Intervention dominant	85% at €10,000 (£12,126)
Williams (2019) [33] *N*=159	Healthy lifestyle intervention	Australia	6	−AUD614 (−3,094, 245) (−£361 (−1,819, 144))	0.02 (−0.00, 0.04)	Intervention dominant	90% at AUD17,000 (£9,993), and 96% at A $67,000 (£39,384)
Herman (2017) [24] N=101	Mindfulness-Based Stress Reduction	USA	12	−USD724 (−4,386, 2,778) (−£729 (−4,419, 2,799))	0.034 (0.008, 0.060)	Intervention dominant	90% at USD50,000 (£50,347)
Herman (2017) [24] *N*=98	CBT	USA	12	USD125 (−4,103, 4,347) (£126 (−4,134, 4,380))	0.041 (0.015, 0.067)	US $3,049 (£2,839)	81% at USD50,000 (£50,347)
Ratcliffe (2006) [37] *N*=160	Traditional Chinese Acupuncture	UK	24	£471 (£779)	0.027 (−0.056, 0.110)	£4,241 (£7,011)	90% at £20,000 (£33,060)
Skonnord (2022) [39] *N*=81	Acupuncture	Norway	12	−USD538 (−£459)	0.0350	USD15,389 (£11,510)	74.1% at NOK 275,000 (£24,704)
Cherkin (2011) [41] *N*=132^†^	Structural massage	USA	12	−USD244 (−978, 722) (−£254 (−1,018, 752))	0.017 (−0.01, 0.04)	USD14,022 (£14,598)	NA
Kongstad (2024) [28] *N*=149	App based self-management support	Denmark	9	€589 (−2,120, 3,298) (£461 (−1,657, 2,578))	0.03 (0.01, 0.05)	€18,821 (£14,714)	~50% at ~€23,250 (£18,181)
Cherkin (2001) [45] *N*=78^†^	Therapeutic massage	USA	12	−USD197 (−649, 296) (−£260 (−856, 391))	0.015 (0.00, 0.03)	USD13,133 (£17,328)	NA
Cherkin (2009) [44] *N*=158^†^	Standardised acupuncture	USA	12	−USD402 (−1,208, 447) (−£372 (−1,118, 414))	0.021 (0.00, 0.04)	USD19,143 (£17,725)	NA
Hurwitz (2002) [42] *N*=170^†^	Physiotherapy	USA	12	−USD223 (−1,777, 1,658) (−£288 (−2,297, 2,143))	0.015 (−0.03, 0.05)	USD15,067 (£19,475)	NA
Cherkin (2011) [41] *N*=132^†^	Relaxation massage	USA	12	−USD491 (−1,195, 431) (−£511 (−1,244, 449))	0.023 (0.00, 0.04)	USD20,982 (£21,833)	NA
Cherkin (2009) [44] *N*=157^†^	Individualised acupuncture	USA	12	−USD592 (−1,430, 367) (−£548 (−1,324, 340))	0.025 (0.00, 0.05)	USD23,680 (£21,921)	NA
Hurwitz (2002) [42] *N*=169^†^	SMT	USA	12	−USD142 (−1,655, 1,362) (−£184 (−2,139, 1,760))	0.008 (−0.03, 0.04)	USD18,684 (£24,142)	NA
Von Korff (2005) [43] *N*=119^†^	Multidisciplinary intervention	USA	12	−USD369 (−1,168, 563) (−£442 (−1,400, 675))	0.015 (−0.01, 0.04)	USD24,600 (£29,487)	NA
Haake (2007) [46] *N*=387^†^	Acupuncture	German	6	−USD1,295 (−2,191, −58) (−£1,161 (−1,965, −52))	0.029 (0.00, 0.05)	USD44,655 (£40,023)	NA
Cherkin (2001) [45] *N*=94^†^	Traditional Chinese Medical acupuncture	USA	12	USD302 (−304, 875) (£399 (−401, 1,156))	0.004 (−0.01, 0.02)	USD75,500 (£99,571)	NA
**Payer perspective**							
Darlow (2019) [22] *N*=126	GP training in fear reduction	New Zealand	6	−NZD422 (−895, to 50) (−£183 (−389, 22))	0.005 (−0.014, 0.024)	Intervention dominant	~85% at NZD55,615 (£24,006)
Herman (2026) [40] *N*=269	Enhanced Acupuncture	USA	12	−USD421 (−2,707, to 1,249) (−£340 (−2,184, 1,008))	0.037 (0.013, 0.062)	Intervention dominant	NA
Hurwitz (2002) [42] *N*=170^†^	Physiotherapy	USA	12	−USD13 (−1,108, 1,130) (−£17 (−1,432, 1,460))	0.015 (−0.03, 0.05)	USD878 (£1,135)	NA
Cherkin (2011) [41] *N*=132^†^	Structural massage	USA	12	USD54 (−447, 658) (£56 (−465, 685))	0.017 (−0.01, 0.04)	USD3,103 (£3,233)	NA
Cherkin (2011) [41] *N*=132^†^	Relaxation massage	USA	12	−USD116 (−566, 529) (−£121 (−590, 551))	0.023 (0.00, 0.04)	USD4,957 (£5,164)	NA
Cherkin (2009) [44] *N*=158^†^	Standardised acupuncture	USA	12	−USD117 (−614, 481) (−£108 (−568, 445))	0.021 (0.00, 0.04)	USD5,571 (£5,157)	NA
Cherkin (2001) [45] *N*=78^†^	Therapeutic massage	USA	12	USD69 (−202, 393) (£91 (−267, 519))	0.015 (0.00, 0.03)	USD4,600 (£6,066)	NA
Hurwitz (2002) [42] *N*=169^†^	Spinal manipulation	USA	12	−USD40 (−1,012, 935) (−£52 (−1,308, 1,208))	0.008 (−0.03, 0.04)	USD5,263 (£6,801)	NA
Cherkin (2009) [44] *N*=157^†^	Individualised acupuncture	USA	12	−USD202 (−708, 496) (−£187 (−655, 459))	0.025 (0.00, 0.05)	USD8,080 (£7,480)	NA
Hochheim (2021) [25] *N*=112	Multidisciplinary biopsychosocial rehabilitation	Germany	24	€781 (£807)	0.087	€8,296 (£8,577)	64% at €20,000 (£20,677) 74% at €30,000 (£31,016)
Von Korff (2005) [43] *N*=119^†^	Multidisciplinary intervention	USA	24	−USD172 (−699, 450) (−£187 (−761, 491))	0.015 (−0.01, 0.04)	USD11,466 (£12,479)	NA
Haake (2007) [46] *N*=387^†^	Acupuncture	German	6	−USD877 (−1,425, −28) (−£786 (−1,277, −25))	0.029 (0.00, 0.05)	USD30,241 (£27,105)	NA
Herman (2026) [40] *N*=265	Standard Acupuncture	USA	12	USD843 (−681, 2,497) (£680 (−549, 2,014))	0.014 (−0.014, 0.043)	USD58,528 (£47,215)	NA
Cherkin (2001) [45] *N*=94^†^	Traditional Chinese Medical acupuncture	USA	12	USD328 (−83, 691) (£433 (−110, 912))	0.004 (−0.01, 0.02)	USD82,000 (£108,124)	NA
**Modelling studies (payer perspective)**							
Verhaeghe (2018) [32] *N*=1,298	Osteopathy	Belgium	12	−€163 (−260, −49.1) (−£164 (−262, −49))	0.06 (−0.06, 0.12)	Intervention dominant	NA
Kim (2010) [36] *N*=2,982	Acupuncture+ usual care	South Korea	3	KRW3,447,840 (£2,425)	4.24	KRM 3,421,394 (£572)	72.3% at KRW 20 million (£14,161)

Inflation: All costs provided in brackets were first brought to 2024 levels using the specific country’s Organisation for Economic Co-operation and Development (OECD) Consumer Price Index (CPI) relative to the year the study was conducted.

Currency: Once in 2024 local prices, the figures were divided by the 2024 OECD Purchasing Power Parity (PPP) for Gross Domestic Product (GDP) rate for that country to convert them into 2024 British Pounds (£).

Intervention is cost-effective when value >0.

“-” minus dollar values indicates the intervention is less costly compared to usual care.

*95% credibility interval.

† Data extracted from Herman (2020) [79], assuming a ‘typical’ mix of chronic LBP patients.

Abbreviations: QALY, Quality-adjusted life year; WTP, willingness to pay; GP, general practitioner; CBT, cognitive behavioural therapy; SMT, spinal manipulative therapy; NA, Not Applicable; UK, United Kingdom; USA, United States of America; AUD, Australian dollars; KRW, Korean Won; NOK, Norwegian kroners; NZD, New Zealand dollars; USD, American dollars; £, British Pounds; €, Euros.

### Methodology quality

Economic evaluation studies were in general of high methodological quality. Eight of the 19 CHEC checklist criteria were met by all trial-based economic evaluations (e.g., clear description of the study population, research question, appropriateness of study design, and time horizon). The least comprehensively reported study assessed with the CHEC checklist was by Johnson and colleagues [38] meeting 13 of the 19 checklist criteria (Table 4). Using the Philips checklist for the three modelling studies, the least comprehensively reported study was by Herman and colleagues (2020) [35] which met only 2 of the 57 item criteria. The most comprehensively reported study was by Kim and colleagues (2010) [36] which met the criteria for 11 of the 57 items (see Table 5).

**Table 4:** Quality assessment scores of within trial economic evaluation studies using the CHEC-list criteria.

CHEC-list item	**Economic Evaluation Study (RCT-based)**																	
		Chuang (2012)	Darlow (2019)	Geraghty (2024)	Herman (2026)	Herman (2017)	Hochheim (2021)	Hollinghurst (2008)	Johnson (2007)	Kent (2023)	Kongstad (2024)	Lamb (2010)	Lambeek (2010)	Ratcliffe (2006)	Skonnord (2022)	Suman (2019)	UK BEAM (2005)	Williams (2019)
	1	Y	Y	Y	Y	Y	Y	Y	Y	Y	Y	Y	Y	N	Y	Y	Y	Y
	2	Y	Y	Y	Y	Y	Y	Y	Y	Y	Y	Y	Y	N	N	Y	N	Y
	3	Y	Y	Y	Y	Y	Y	Y	Y	Y	Y	Y	Y	Y	Y	Y	Y	Y
	4	Y	Y	Y	Y	Y	Y	Y	Y	Y	Y	Y	Y	Y	Y	Y	Y	Y
	5	Y	Y	Y	Y	Y	Y	Y	Y	Y	Y	Y	Y	Y	Y	Y	Y	Y
	6	Y	Y	Y	Y	Y	Y	Y	N	Y	Y	Y	Y	N	Y	Y	N	N
	7	Y	Y	Y	Y	Y	Y	Y	N	Y	Y	Y	Y	Y	Y	Y	N	Y
	8	Y	Y	Y	Y	Y	Y	Y	Y	N	Y	Y	Y	Y	Y	Y	Y	Y
	9	Y	N	Y	Y	Y	Y	Y	Y	Y	Y	Y	Y	Y	Y	Y	Y	Y
	10	Y	Y	Y	Y	Y	Y	Y	Y	Y	Y	Y	Y	Y	Y	Y	Y	Y
	11	Y	Y	Y	Y	Y	Y	Y	Y	Y	Y	Y	Y	Y	Y	Y	Y	Y
	12	Y	Y	Y	Y	Y	Y	Y	Y	Y	Y	Y	Y	Y	Y	Y	Y	Y
	13	Y	Y	Y	Y	Y	Y	Y	Y	Y	Y	Y	Y	Y	Y	Y	Y	Y
	14	Y	N	Y	N	Y	Y	Y	N	N	Y	Y	Y	Y	N	Y	Y	Y
	15	Y	Y	Y	Y	Y	Y	Y	N	Y	Y	Y	Y	Y	N	Y	Y	Y
	16	Y	Y	Y	Y	Y	Y	Y	N	Y	Y	Y	Y	Y	Y	Y	Y	Y
	17	Y	N	N	Y	Y	Y	Y	Y	Y	Y	Y	Y	Y	Y	Y	Y	Y
	18	Y	Y	Y	Y	Y	Y	Y	Y	Y	Y	Y	Y	Y	Y	Y	Y	Y
	19	Y	Y	Y	Y	Y	Y	Y	N	Y	Y	Y	Y	Y	Y	Y	Y	Y

RCT, randomised controlled trial; Y, yes; N, No.

**Table 5:** Quality assessment scores of modelling studies using the Phillips’ quality assessment checklist.

Philips’ criteria		Herman (2020)	Kim (2010)	Verhaeghe (2018)
**STRUCTURE**				
1.	Is there a clear statement of the decision problem?	N	Y	Y
2.	Is the objective of the model specified and consistent with the stated decision problem?	UNC	UNC	UNC
3.	Is the primary decision maker specified?	N	N	N
4.	Is the perspective of the model stated clearly?	P	Y	Y
5.	Are the model inputs consistent with the stated perspective?	UNC	Y	P
6.	Has the scope of the model been stated and justified?	N	UNC	P
7.	Are the outcomes of the model consistent with the perspective, scope and overall objective of the model?	N	Y	Y
8.	Is the structure of the model consistent with a coherent theory of the health condition under evaluation?	UNC	UNC	UNC
9.	Are the sources of the data used to develop the structure of the model specified?	N	N	N
10.	Are the causal relationships described by the model structure justified appropriately?	N	N	N
11.	Are the structural assumptions transparent and justified?	N	N	N
12.	Are the structural assumptions reasonable given the overall objective, perspective and scope of the model?	N	N	N
13.	Is there a clear definition of the options under evaluation?	N	P	Y
14.	Have all feasible and practical options been evaluated?	N	N	N
15.	Is there justification for the exclusion of feasible options?	N	N	N
16.	Is the chosen model type appropriate given the decision problem and specified casual relationships within the model?	UNC	UNC	UNC
17.	Is the time horizon of the model sufficient to reflect all important differences between the options?	Y	Y	Y
18.	Are the time horizon of the model, the duration of treatment and the duration of treatment described and justified?	P	Y	P
19.	Do the disease states (state transition model) or the pathways (decision tree model) reflect the underlying biological process of the disease in question and the impact of interventions?	UNC	Y	Y
20.	Is the cycle length defined and justified in terms of the natural history of disease?	P	P	P
**DATA**				
21.	Are the data identification methods transparent and appropriate given the objectives of the model?	N	P	P
22.	Where choices have been made between data sources are these justified appropriately?	N	UNC	UNC
23.	Has particular attention been paid to identifying data for the important parameters of the model?	N	P	P
24.	Has the quality of the data been assessed appropriately?	N	N	N
25.	Where expert opinion has been used are the methods described and justified?	NA	NA	NA
26.	Is the data modelling methodology based on justifiable statistical and epidemiological techniques?	N	N	N
27.	Is the choice of baseline data described and justified?	N	UNC	UNC
28.	Are transition probabilities calculated appropriately?	P	P	NA
29.	Has a half-cycle correction been applied to both costs and outcomes?	N	N	N
30.	If not, has the omission been justified?	N	N	N
31.	If relative treatment effects have been derived from trial data, have they been synthesised using appropriate techniques?	P	Y	P
32.	Have the methods and assumptions used to extrapolate short-term results to final outcomes been documented and justified?	N	N	N
33.	Have alternative extrapolation assumptions been explored through sensitivity analysis?	N	N	N
34.	Have assumptions regarding the continuing effect of treatment once treatment is complete been documented and justified?	N	N	N
35.	Have alternative assumptions regarding the continuing effect of treatment been explored through sensitivity analysis	N	N	N
36.	Are the costs incorporated into the model justified?	N	Y	Y
37.	Has the source for all costs been described?	N	Y	Y
38.	Have discount rates been described and justified given the target decision maker?	N/A	Y	Y
39.	Are the utilities incorporated into the model appropriate?	UNC	UNC	Y
40.	Is the source of utility weights referenced?	N	Y	UNC
41.	Are the methods of derivation for the utility weights justified?	N	N	N
42.	Have all data incorporated into the model been described and referenced in sufficient detail?	Y	P	P
43.	Has the use of mutually inconsistent data been justified (i.e., are assumptions and choices appropriate?)	UNC	UNC	UNC
44.	Is the process of data incorporation transparent?	N	UNC	UNC
45.	If data have been incorporated as distributions, has the choice of distributions for each parameter been described and justified?	P	P	P
46.	If data have been incorporated as distributions, is it clear that second-order uncertainty is reflected?	P	P	P
47.	Have the four principal types of uncertainty been addressed?	N	N	N
48.	If not, has the omission of particular forms of uncertainty been justified?	N	N	N
49.	Have methodological uncertainties been addressed by running alternative versions of the model with different methodological assumptions?	N	N	N
50.	Is there evidence that structural uncertainties have been addressed via sensitivity analysis?	N	N	N
51.	Has heterogeneity been dealt with by running the model separately for different sub-groups?	N	P	N
52.	Are the methods of assessment of parameter uncertainty appropriate?	N	P	P
53.	If data are incorporated as point estimates, are the ranges used for sensitivity analysis stated clearly and justified?	UNC	UNC	UNC
54.	Is there evidence that the mathematical logic of the model has been tested thoroughly before use?	N	UNC	UNC
55.	Are any counterintuitive results from the model explained and justified?	N	N	N
56.	If the model has been calibrated against independent data, have any differences been explained and justified?	N	Y	N
57.	Have the results been compared with those of previous models and any differences in results explained?	N	P	P
N		38	21	23
Y		2	11	10
P		7	11	12
UNC		8	13	10
NA		2	1	2

N, No; Y, Yes; P, Partial; UNC, Unclear; NA, Not Applicable.

### Primary outcome

We report the main results based on the primary outcome, incremental cost-effectiveness ratio (ICER) expressed as cost/QALY in Table 3, sorted by perspective and ranked in order of cost-effectiveness. From the within-trial economic analyses, 6 single-modality interventions (yoga, acupuncture, cognitive behavioural therapy, self-management support, fear reduction education and mindfulness stress reduction) and 2 multimodal interventions (advice plus graded activity at the workplace, healthy lifestyle (diet plus physical activity)) were more effective and less costly than usual care; i.e., intervention dominated control from one or more perspectives (Table 3) [21–24, 27, 30, 31, 33]. In one modelling study, osteopathy was also found to dominate usual care [32]. In only one comparison, a trial of massage therapy, did the control intervention dominate the intervention.

From a healthcare perspective in 14/18 comparisons (e.g., exercise plus cognitive therapy, exercise alone, spinal manipulation) the intervention was cost-effective at our notional WTP threshold of £20,000. Of the nine comparisons reporting the probability of cost-effectiveness, levels of certainty ranged from 27% at £14,697 for standard acupuncture to 98% at £18,181 app-based self-management support [28, 40].

From a societal perspective in 15/19 comparisons (e.g., therapeutic and structural massage, physiotherapy) the intervention was cost effective at our notional WTP threshold of £20,000. The probability of an intervention being cost-effective was reported in nine comparisons and levels of certainty ranged from ~50% at £18,181 for app-based self-management to 96% at £39,384 for a healthy lifestyle intervention [28, 33].

From a payer perspective, in 11/14 trial-based comparisons (e.g., physiotherapy, massage, multidisciplinary intervention, spinal manipulation), the intervention appears to be cost-effective at our notional WTP threshold of £20,000. Of the two comparisons for which the probability of an intervention being cost-effective was reported, levels of certainty ranged from 64% at £17,686 for MBR to ~85%% at £24,006 for GP training in fear reduction [22, 25].

Both studies modelling cost-effectiveness of osteopathy and acupuncture also found a cost per QALY below our notional WTP threshold of £20,000 from a payer perspective [32, 36]. Only one study reported the net monetary benefit of the evaluated intervention. Darlow and colleagues [22] showed that adding fear reduction education to general practice care of LBP yields NZ$542 (95% CI −678, 1,762) (£281 [95%CI −411, 1,067]) more in value than it costs compared with general practitioner (GP) care alone, from a healthcare perspective, NZ$609 (95% CI −1,385, 2,603) (£315 [95%CI −839, 1,577]) from a societal perspective and NZ$-695 (95%CI −496, 1,885) (£ −360 [95%CI −301, 1,142]) from a payer perspective at a WTP threshold of NZ$ = 55,615 (£32,740).

### Secondary outcomes

We summarise the economic costs related to secondary outcomes of direct and indirect costs in S5 File. All studies reported total costs, such as interventional costs of healthcare. Nine studies reported indirect costs (64%) [21–24, 27, 28, 30, 31, 33, 47–76]. Lost productivity and absenteeism were the most common individual indirect costs reported (detailed in S4 File—Table).

## Discussion

For those interventions recommended in selected current LBP guidelines for which health economic analyses are available, our findings show a consistent pattern that many non-pharmacologic and non-surgical single-modality and multimodal, clinician-delivered interventions represent good value for money from whichever perspective has been assessed. Compared to usual care, yoga, acupuncture, cognitive interventions, fear and stress reduction therapies, and self-management support are potentially cost-saving, single-modality treatments shown to be more effective and reduce overall costs (e.g., £2,671 reduction of societal costs for cognitive functional therapy; £396 reduction of healthcare costs for acupuncture). Moreover, multimodal, combined therapies, such as healthy lifestyle promotion (diet plus physical activity), and advice plus graded activity, were shown to be more effective and to incur fewer costs than usual medical care (i.e., £7,315 reduction in societal costs for workplace advice and graded activity, and £361 reduction for diet plus exercise). Other combined therapies, including exercise and spinal manipulation, exercise and cognitive therapy, and multidisciplinary rehabilitation are also cost-effective, compared to usual care, at least from societal and payer perspectives. Massage therapy and acupuncture were commonly evaluated across included studies, however, with inconsistent results, suggesting their cost-effectiveness varies substantially with context, perspective and mode of delivery. Given the high level of heterogeneity across studies in terms of perspectives, comparators, methodological quality of original randomised trial data and country settings, these results should be interpreted with caution.

Previous systematic reviews on the cost-effectiveness of treatments for LBP have been specific to individual therapies [7, 8, 77], with no comprehensive review of internationally recommended, guideline-concordant recommendations for managing acute and chronic LBP. Since the last review was published in 2019, 9 new economic evaluations meeting our eligibility criteria have been published and included in our main results. Our review, however, has identified that interventions that aim at reducing fear and stress related to LBP, as well as clinician-delivered mind-body therapies, such as yoga, acupuncture, and cognitive behavioural therapy, can be potentially cost-saving, as they are both more effective and less costly than usual medical care. These interventions are still far from being routinely implemented or commissioned in most countries. Future efforts should focus on elucidating and addressing the factors contributing to this gap and to inform strategies aimed at reducing it.

The evidence generated in this systematic review highlights that multiple guideline-recommended treatments for LBP offer good value for money, over usual care, with many offering potential cost-savings to society, and healthcare system. Efforts must now focus on implementing these interventions at scale to ensure their benefits are fully realised. Our findings also highlight that cost-related recommendations for managing LBP in low- to middle-income countries remain limited, as generalisations from high-income countries, although appropriate in some components, are unlikely to be relevant. Healthcare resources can be limited in some settings, contexts and countries, and hence, policymakers and clinicians should tailor and optimise treatment approaches with dynamic, local considerations, including options to reduce inequities in access to care, e.g., through harnessing digitally-enabled service models.

Undertaking a thorough, unrestricted search for studies of guideline-recommended interventions to manage LBP compared to usual care provides the current context and landscape of the topic. The internationally recognised guidelines used in this review will likely be known and used by clinicians. Although we were unable to conduct a meta-analysis due to the heterogeneity of included study designs and jurisdictions, this systematic review is the first to our knowledge to summarise direct and indirect costs of guideline-recommended interventions used to manage LBP. We observed heterogeneity in the data, including how cost-effectiveness was measured and a lack of standardisation in reporting costs. Studies reporting direct costs included intervention or total costs but infrequently reported community services costs, out-of-pocket costs, and indirect costs. Moreover, costs are context-specific and may have limited generalisability beyond the healthcare system in which they were collected and reported. Furthermore, the current evidence regarding specific LBP populations (e.g., sciatica) was not included in the current report. Standard GRADE criteria (e.g., blinding, directness) do not adequately capture the core drivers of economic uncertainty, such as model structure, parameter inputs, and time-horizon assumptions. Consequently, to ensure a technically rigorous evaluation, we deviated from the protocol by omitting GRADE and instead utilising established, specific health economic appraisal tools (including the CHEC checklist) to assess evidence quality and methodological certainty. Pre-specified subgroup stratifications, as well as distinct sensitivity analyses (e.g., restricting cohorts to chronic LBP), could not be executed due to an inadequate volume of split-sample primary data. Finally, to ensure a consistent baseline benchmark, this review restricted eligible comparators to usual care, sham/placebo, or no treatment. While multi-arm active economic evaluations offer direct comparative utility within localised contexts, their inclusion in a broad global synthesis introduces further heterogeneity and limits the relevance of the results to policy and service model considerations, particularly in the context of financing and commissioning decisions and decisions about which interventions may be appropriately considered in Universal Health Coverage essential packages. Restricting the comparator to a standard baseline allows for a clearer evaluation of the absolute incremental economic value of guideline-recommended interventions relative to routine clinical practice or no care.

The high burden, costs and disability that LBP places on society, impacting millions worldwide, make the management of LBP a global priority. The lack of evidence related to cost-effectiveness in managing LBP from the perspective of low- to middle-income countries highlights a clear opportunity for future, high-quality research [78]. While standardising the cost-effectiveness measures (e.g., ICER) may assist consistency in future studies, extending the reporting of costs beyond intervention and total costs (e.g., lost productivity and community costs) would enable more accurate measures of burden. Finally, we have identified the urgent need for cost-effectiveness data on pharmacological and surgical interventions for LBP, given how commonly these are accessed and funded globally.

## Supporting information

S1 Checklist PRISMA checklist. Page MJ, McKenzie JE, Bossuyt PM, Boutron I, Hoffmann TC, Mulrow CD, et al. The PRISMA 2020 statement: an updated guideline for reporting systematic reviews. MetaArXiv. 2020, September 14. https://doi.org/10.31222/osf.io/v7gm2. For more information, visit: https://www.prisma-statement.org.
(DOCX)

S1 File Search strategy. (DOCX)

S2 File Eligible interventions to manage low back pain. (DOCX)

S3 File Quality assessment and scoring. (DOCX)

S4 File Direct and indirect costs in included studies. (DOCX)

S5 File Studies excluded from this report and main reason for exclusion. (DOCX)

## Abbreviations

BEST: Back Skills Training
CBT: Cognitive Behavioral Therapy
CEA: Cost-effectiveness Analysis Registry
CFT: Cognitive Functional Therapy
CHEC: Consensus Health Economics Criteria
CHEERS: Consolidated Health Economics Evaluation Reporting Standards
CI: confidence intervals
CPI: Consumer Price Index
FREE: Fear Reduction Exercised Early
GBP: Great British Pound
GDP: Gross Domestic Product
GP: general practitioner
GRADE: Grading of Recommendations Assessment, Development and Evaluation
HTA: Health Technology Assessment
ICER: incremental cost-effectiveness ratio
LBP: low back pain
MBR: multidisciplinary biopsychosocial rehabilitation
MBSR: Mindfulness-Based Stress Reduction
NASS: North American Spine Society
NICE: National Institute for Health and Care Excellence
PPP: Purchasing Power Parity
PRISMA: Preferred Reporting Items for Systematic reviews and Meta-Analyses
QALY: quality-adjusted life year
USD: United States Dollar
WHO: World Health Organization
WTP: Willingness to pay
