# Supplementary material for: Cost-effectiveness of recommended interventions for managing low back pain: A systematic review
Source: PLoS Med. 2026 Oct 27;23(10):e1005273. doi: 10.1371/journal.pmed.1005273 (PMC13712551; doi:10.1371/journal.pmed.1005273)
Supplement: Supplementary file 2 [file pmed.1005273.s002.docx]

**Supplementary material 2: Search strategy**

**Databases**

- MEDLINE (OvidSP)
- EMBASE (OvidSP)
- PsycINFO (OvidSP)
- Cochrane Central Register of Controlled Trials (CENTRAL) (OvidSP)
- CINAHL Complete (EBSCOhost)
- EconLit with full text (EBSCOhost)
- The Tufts Cost-Effectiveness Analysis Registry (CEA)
- NHS Economic Evaluation Database (OvidSP)
- Health Technology Assessment (HTA) (OvidSP)
- International Pharmaceutical Abstracts (OvidSP)

The search strategy was adapted for each database.

**Search strategy for OvidSP databases**

((randomised controlled trial) OR (randomized controlled trial) OR (controlled clinical trial) OR (comparative study) OR (clinical trial) OR (pragmatic clinical trial) OR (cross-over stud$) OR random OR (cross over) OR (cross-over) OR (crossover) OR (double adj blind$) OR (single adj blind$) OR (before and after) OR (case control) OR (cohort study) OR (quasi experiment$) OR (time series) OR placebo OR (usual care) OR (no intervention) OR trial OR groups OR observational OR modelling)

AND Adult AND ((low back pain) OR (lower back pain) OR (back pain) OR (dorsalgia) OR backache OR (back ache) OR spondylosis OR (back disorder) OR lumbago OR coccydynia OR sciatica OR (sciatic neuropathy) OR radiculopathy OR (radicular pain)) OR ((back OR lumb$) adj3 (pain OR radicul$ OR polyradicul$)) AND (intervention OR management OR treatment OR strategies OR education OR advice OR exercise OR (physical activity) OR (physical therapy) OR (manual therapy) OR manipulation OR (spinal manipulative therapy) or manipulation or mobilisation OR massage OR (soft tissue therapy) OR heat OR (heat wrap) or (heat pack) OR (thermal therap$) OR ((behavio* OR cognitive OR psycho* OR social) adj (therap* OR treatment)) OR (cognitive functional therap$) OR (operant therapy) OR (operant conditioning) OR counseling OR counselling OR psychotherapy OR multidisciplinar$ OR interdisciplinar$ OR multiprofessional$ OR multimodal$ OR multicomponent$ OR multi-disciplinar$ OR inter-disciplinar$ OR multi-professional$ OR multi-modal$ OR multi-component$ OR rehabilitation OR (functional restoration) OR (occupational therapy) OR (mobility aid) or (physical aid) OR (walking aid) OR (walking frame OR stick OR cane) OR crutch OR (nonsteroidal antiinflammatory drugs) OR (non steroidal anti inflammatory drugs) OR NSAIDs OR ((cyclooxygenase OR cyclo oxygenase) adj3 inhibitor) OR opioid OR opiate OR narcotic OR (cayenne pepper) OR (capsicum annuum) OR (epidural steroid injection) OR (steroid injection) OR steroid OR corticosteroid OR glucocorticosteroid OR decompression or (radiofrequency denervation) OR (radio frequency) OR (radiofrequency) OR (radiofrequency ablation)) AND (economic OR cost OR costs OR price OR price OR expenditure OR value OR valuation OR budget OR investment OR (net benefit) OR (net-benefit) OR saving OR profit OR financial OR invest OR investment OR fiscal OR monetary OR money OR dollar OR (willingness to pay) OR (willingness-to-pay) OR (willing to pay) OR charge OR charges OR pay OR payment OR fee OR fees OR spending OR unit cost OR (cost minimize) OR (cost minimise) OR net-cost OR net-costs OR (net cost) OR (net costs) OR (cost analysis) OR (cost comparison) OR (cost-effectiveness) OR (cost effectiveness) OR (cost-utility) OR (cost utility) OR (cost-benefit) OR (cost benefit) OR (economic evaluation) OR (health economics) OR (health resource allocation) OR (decision analysis) OR QALY OR (quality adjusted life year)) AND (human NOT animal)

**The Tufts Cost-Effectiveness Analysis Registry (CEA)**

- Back pain, low back pain in keyword search
