# Supplementary material for: Cost-effectiveness of recommended interventions for managing low back pain: A systematic review
Source: PLoS Med. 2026 Oct 27;23(10):e1005273. doi: 10.1371/journal.pmed.1005273 (PMC13712551; doi:10.1371/journal.pmed.1005273)
Supplement: Supplementary file 3 [file pmed.1005273.s003.docx]

**Supplementary material 3: Eligible interventions to manage low back pain**

Interventions to manage LBP recommended in favour of use by at least one of the following guidelines: World Health Organisation (WHO 2023), National Institute for Health and Care Excellence (NICE 2020), and North American Spine Society (NASS 2020).

Interventions in grey rows were not included in the review.

| THERAPY | GUIDELINE AND TARGET POPULATION^^^ | | |
| --- | --- | --- | --- |
|  | **NICE**  **2020 update** | **WHO 2023** | **NASS 2020** |
|  | LBP ± leg pain | cLBP ± leg pain | NSLBP |
| NON-PHARMACOLOGICAL |  |  |  |
| Educational/advice strategies | In favour | In favour of structured and standardised education | ⎯ (conflicting evidence)  In favour for back schools |
| Exercise/physical activity | In favour for group exercise | In favour for structured exercise programs | In favour for  McKensie for cLBP  Yoga for mild cLBP  Aerobic for NSLBP |
| Traction | Against | Against | Against |
| Manual therapy (manipulation, mobilisation) | In favour (when in treatment package^#^) | In favour (spinal manipulative therapy) | In favour (spinal manipulative therapy) |
| Massage/soft tissue therapy | In favour (when in treatment package^#^) | In favour | ⎯ (conflicting evidence for acupressure)  ⎯ (Massage) |
| Acupuncture/needling | Against | In favour | ⎯ (insufficient evidence for dry needling)  ⎯ (conflicting evidence for acupuncture) |
| Electrotherapies | Against | Against | Against for ultrasound (not effective)  ⎯ (conflicting evidence for TENS) |
| Heat | ⎯ | ⎯ | In favour |
| Psychosocial/psychological therapies  Cognitive behavioural therapy  Mindfulness, respondent therapy  Operant therapy | In favour^#^  In favour^#^  ⎯  ⎯ | ⎯  In favour  ⎯  In favour | ⎯  In favour^#^  ⎯  ⎯ |
| Return to work programmes | In favour | – | In favour (to consider work hardening) |
| Assistive products for the lower back | Against for belts, corsets, orthotics, rocker shoes | Against for lumbar braces, belts, supports, orthotics | ⎯ (conflicting evidence for braces) |
| Mobility assistive products | ⎯ | In favour | ⎯ |
| Multi-component interventions | In favour for combined physical and psychological programmes | In favour (least two domains of  the biopsychosocial model of chronic pain (biological, psychological, social)  ⎯ non-pharmacological weight loss multi-component interventions | In favour for cognitive behavioural therapy + physical  therapy |
| PHARMACOLOGICAL |  |  |  |
| Paracetamol (acetaminophen) | Against as single ingredient | ⎯ | ⎯ |
| NSAIDs | In favour for judicious use | In favour | In favour for non-selective NSAIDs  ⎯ insufficient evidence for selective NSAIDs |
| Opioid analgesics | Judicious use of weak opioids if needed in aLBP e.g. NSAIDs contraindicated. In combination with paracetamol permitted  Against for chronic sciatica | Against | Judicious use |
| Anti-convulsants, gabapentinoids | Against | Against | ⎯ |
| Benzodiazepines | Against | ⎯ | ⎯ |
| Anti-depressants | Against | Against | Against |
| Oral steroids | Against (corticosteroids) | Against (glucocorticoids) | Against (not effective) |
| Skeletal muscle relaxants | ⎯ | Against | ⎯ |
| Topical cayenne pepper | ⎯ | In favour | In favour (topical capsicum) |
| Cannabinoids | ⎯ | ⎯ | ⎯ |
| Lidocaine patch | ⎯ | ⎯ | ⎯ (insufficient evidence) |
| Other – non-drug, e.g. herbal | ⎯ | ⎯ / Against  (including weight loss interventions) | ⎯ (insufficient evidence for Vitamin D) |
| INVASIVE, NON-SURGICAL INTERVENTIONS | | | |
| Steroid injections | Against for spinal injections for back pain | ⎯ | ⎯ (insufficient evidence for caudal and intralaminar epidural steroid injections)  ⎯ (insufficient evidence for zygapophyseal joint steroid injection)  In favour for intradiscal steroids for patients with Modic changes  Against (not effective for IV steroids) |
|  | ⎯ | ⎯ | In favour for intra-articular injection for SIJ pain |
| Local anaesthetic injection | Against for spinal injections for back pain  In favour for acute & severe sciatica | Against  Against | ⎯  ⎯ |
| Platelet rich plasma, intradiscal bone marrow, intradiscal steroids | ⎯ | ⎯ | ⎯ (insufficient evidence for discogenic LBP) |
| SURGICAL INTERVENTIONS |  |  |  |
| Decompression | Only consider for unresolved sciatica | ⎯ | ⎯ |
| Fusion | Against, unless for an RCT participation | ⎯ | ⎯ (insufficient evidence for particular fusion technique) |
| Disc replacement | Against | ⎯ | ⎯ |
| Radiofrequency denervation | In favour to potentially consider for cLBP | ⎯ | In favour for thermal radiofrequency ablation for LBP from zygapophyseal joints  In favour for cooled radiofrequency ablation for SIJ pain |
| Spinal cord stimulator | ⎯ | ⎯ | ⎯ (insufficient evidence) |

**Table A1: Summary of guideline recommendations to manage low back pain.**

**Abbreviations**

aLBP = acute LBP; cLBP = Chronic Low Back Pain; LBP = Low Back Pain; NSAIDs = Non-Steroidal anti-Inflammatory Drugs; RCT = Randomised Clinical Trial; SIJ = Sacroiliac Joint; TENS = Transcutaneous Electrical Nerve Stimulation.

**Footnotes**

\# in treatment package that includes exercise with or without psychological therapy.

⎯ Not mentioned, or no position stated; reasons provided if available, e.g. no recommendation because of insufficient evidence.

**^ Guidelines**

**National Institute for Health and Care Excellence (NICE).** *Low back pain and sciatica in over 16s: assessment and management*. 2020. Available at: [www.nice.org.uk/guidance/ng59](http://www.nice.org.uk/guidance/ng59).

- We followed the guideline recommendations of “for” or “against” a therapy, with any considerations stated. The strength of evidence was considered in determining therapy recommendations by the guideline committee. The guideline does not call out the strength of evidence per recommendation.
- Low back pain, and sciatica is not formally defined in the guideline.

**World Health Organization (WHO).** WHO guideline for non-surgical management of chronic primary low back pain in adults in primary and community care settings. 2023. Available at: <https://www.who.int/publications/i/item/9789240081789>.

- Recommendations are considered “in favour”, “good practice statement”, “against use”, no recommendation or no/insufficient evidence to make a recommendation. The quality of the recommendation is reported within the guideline, e.g. “very-low certainty” following the GRADE assessment.
- Chronic primary low back pain is defined on page xiii, and follows The International Association for the Study of Pain (IASP) definition of chronic primary LBP, “…previously referred to as “non-specific low back pain”, as pain that persists or recurs for longer than three months, and is associated with emotional distress and/or functional disability, and symptoms that cannot be better accounted for by another diagnosis, such as tissue damage or a disease process”.

**North American Spine Society (NASS).** *Evidence-based clinical guidelines for multidisciplinary spine care: diagnosis & treatment of low back* *pain*. 2020. NASS. Illinois. Available at: <https://www.spine.org/Portals/0/assets/downloads/ResearchClinicalCare/Guidelines/LowBackPain.pdf>.

- Therapies are or were not recommended with a “grade of recommendation” being either:
- A: Good evidence (Level I studies with consistent findings) for or against intervention.
- B: Fair evidence (Level II or III studies with consistent findings) for or against intervention.
- C: Poor quality evidence (Level IV or V studies) for or against intervention.
- I: Insufficient or conflicting evidence not allowing a recommendation for or against intervention.

Low back pain as defined on Page 6 as pain of musculoskeletal origin extending from the lowest rib to the gluteal fold that may at times extend as somatic referred pain into the thigh (above the knee).
