# Supplementary material for: Cost-effectiveness of recommended interventions for managing low back pain: A systematic review
Source: PLoS Med. 2026 Oct 27;23(10):e1005273. doi: 10.1371/journal.pmed.1005273 (PMC13712551; doi:10.1371/journal.pmed.1005273)
Supplement: Supplementary file 4 [file pmed.1005273.s004.docx]

**Supplementary material 4: Quality assessment and scoring**

**A. CHEC-list**

Each question was scored as “yes” or “no”. Any unclear domains were scored as “no”.

1. Is the study population clearly described?
2. Are competing alternatives clearly described?
3. Is a well-defined research question posed in answerable form?
4. Is the economic study design appropriate to the stated objective?
5. Is the chosen time horizon appropriate in order to include relevant costs and consequences?
6. Is the actual perspective chosen appropriate?
7. Are all important and relevant costs for each alternative identified?
8. Are all costs measured appropriately in physical units?
9. Are costs valued appropriately?
10. Are all important and relevant outcomes for each alternative identified?
11. Are all outcomes measured appropriately?
12. Are outcomes valued appropriately?
13. Is an incremental analysis of costs and outcomes of alternatives performed?
14. Are all future costs and outcomes discounted appropriately?
15. Are all important variables, whose values are uncertain, appropriately subjected to sensitivity analysis?
16. Do the conclusions follow from the data reported?
17. Does the study discuss the generalizability of the results to other settings and patient/client groups?
18. Does the article indicate that there is no potential conflict of interest of study researcher(s) and funder(s)?
19. Are ethical and distributional issues discussed appropriately?

Evers S, et al. Criteria list for assessment of methodological quality of economic evaluations: Consensus on Health Economic Criteria. *Int J Technol Assess Health Care*. 2005;21(2):240-5.

**B. Phillips’ quality assessment checklist for studies that included an economic model**

Each question was scored as “yes”, “no”, “partial”, “unclear”, or “not applicable”.

| **Philips’ criteria** | |
| --- | --- |
| **STRUCTURE** | |
| 1. | Is there a clear statement of the decision problem? |
| 2. | Is the objective of the model specified and consistent with the stated decision problem? |
| 3. | Is the primary decision maker specified? |
| 4. | Is the perspective of the model stated clearly? |
| 5. | Are the model inputs consistent with the stated perspective? |
| 6. | Has the scope of the model been stated and justified? |
| 7. | Are the outcomes of the model consistent with the perspective, scope and overall objective of the model? |
| 8. | Is the structure of the model consistent with a coherent theory of the health condition under evaluation? |
| 9. | Are the sources of the data used to develop the structure of the model specified? |
| 10. | Are the causal relationships described by the model structure justified appropriately? |
| 11. | Are the structural assumptions transparent and justified? |
| 12. | Are the structural assumptions reasonable given the overall objective, perspective and scope of the model? |
| 13. | Is there a clear definition of the options under evaluation? |
| 14. | Have all feasible and practical options been evaluated? |
| 15. | Is there justification for the exclusion of feasible options? |
| 16. | Is the chosen model type appropriate given the decision problem and specified casual relationships within the model? |
| 17. | Is the time horizon of the model sufficient to reflect all important differences between the options? |
| 18. | Are the time horizon of the model, the duration of treatment and the duration of treatment described and justified? |
| 19. | Do the disease states (state transition model) or the pathways (decision tree model) reflect the underlying biological process of the disease in question and the impact of interventions? |
| 20. | Is the cycle length defined and justified in terms of the natural history of disease? |
| **DATA** | |
| 21. | Are the data identification methods transparent and appropriate given the objectives of the model? |
| 22. | Where choices have been made between data sources are these justified appropriately? |
| 23. | Has particular attention been paid to identifying data for the important parameters of the model? |
| 24. | Has the quality of the data been assessed appropriately? |
| 25. | Where expert opinion has been used are the methods described and justified? |
| 26. | Is the data modelling methodology based on justifiable statistical and epidemiological techniques? |
| 27. | Is the choice of baseline data described and justified? |
| 28. | Are transition probabilities calculated appropriately? |
| 29. | Has a half-cycle correction been applied to both costs and outcomes? |
| 30. | If not, has the omission been justified? |
| 31. | If relative treatment effects have been derived from trial data, have they been synthesised using appropriate techniques? |
| 32. | Have the methods and assumptions used to extrapolate short-term results to final outcomes been documented and justified? |
| 33. | Have alternative extrapolation assumptions been explored through sensitivity analysis? |
| 34. | Have assumptions regarding the continuing effect of treatment once treatment is complete been documented and justified? |
| 35. | Have alternative assumptions regarding the continuing effect of treatment been explored through sensitivity analysis |
| 36. | Are the costs incorporated into the model justified? |
| 37. | Has the source for all costs been described? |
| 38. | Have discount rates been described and justified given the target decision maker? |
| 39. | Are the utilities incorporated into the model appropriate? |
| 40. | Is the source of utility weights referenced? |
| 41. | Are the methods of derivation for the utility weights justified? |
| 42. | Have all data incorporated into the model been described and referenced in sufficient detail? |
| 43. | Has the use of mutually inconsistent data been justified (i.e. are assumptions and choices appropriate?) |
| 44. | Is the process of data incorporation transparent? |
| 45. | If data have been incorporated as distributions, has the choice of distributions for each parameter been described and justified? |
| 46. | If data have been incorporated as distributions, is it clear that second order uncertainty is reflected? |
| 47. | Have the four principal types of uncertainty been addressed? |
| 48. | If not, has the omission of particular forms of uncertainty been justified? |
| 49. | Have methodological uncertainties been addressed by running alternative versions of the model with different methodological assumptions? |
| 50. | Is there evidence that structural uncertainties have been addressed via sensitivity analysis? |
| 51. | Has heterogeneity been dealt with by running the model separately for different sub-groups? |
| 52. | Are the methods of assessment of parameter uncertainty appropriate? |
| 53. | If data are incorporated as point estimates, are the ranges used for sensitivity analysis stated clearly and justified? |
| 54. | Is there evidence that the mathematical logic of the model has been tested thoroughly before use? |
| 55. | Are any counterintuitive results from the model explained and justified? |
| 56. | If the model has been calibrated against independent data, have any differences been explained and justified? |
| 57. | Have the results been compared with those of previous models and any differences in results explained? |
| N- No  Y- Yes  P – Partial  UNC-Unclear  N/A- Not Applicable | |

Philips Z, et al. Good practice guidelines for decision-analytic modelling in health technology assessment: a review and consolidation of quality assessment. *Pharmacoeconomics* 2006;24(4):355-71.
