# Supplementary material for: Cost-effectiveness of recommended interventions for managing low back pain: A systematic review
Source: PLoS Med. 2026 Oct 27;23(10):e1005273. doi: 10.1371/journal.pmed.1005273 (PMC13712551; doi:10.1371/journal.pmed.1005273)
Supplement: Supplementary file 5 [file pmed.1005273.s005.docx]

**Supplementary material 5 Table: Direct and indirect costs in included studies**

| **Study** | **Direct costs**  **(total direct costs, intervention costs, costs with health service use, community service costs &/or out-of-pocket costs)** | **Indirect costs**  **(lost wages, lost productivity, absenteeism)** |
| --- | --- | --- |
| Cherkin 2001 | Treatment cost  - Acupuncture: USD$253  - Massage: USD$139 | NR |
| Cherkin 2009 | Treatment cost  - Individualized Acupuncture: USD$482.38  - Standardized Acupuncture: USD$489.30 | NR |
| Cherkin 2011 | Cost of back pain-related health care (mean (95%CI))  -Relaxation massage: USD$78 (0 to 3,764)  - Structural massage: USD$38 (0 to 1,443)  Treatment cost  - Relaxation massage: USD$461.18  - Structural massage: USD$450.60 | NR |
| Chuang 2012 | Total (societal) costs (mean (SD))  - Yoga: £1,501.1 (£2,550.4)  - Usual care: £2,319.2 (£3,188.1)  *Intervention (mean)*  -Yoga: £292.61  -Usual care: £0  *NHS costs (mean (SD))*  - Yoga: £762.0 (£1,715.2)  - Usual care: £529.7 £ (929.9)  *Private cost (mean (SD))*  - Yoga: £331.2 (£1313)  - Usual care: £ 439.3 (£1,687.4) | *Cost of productivity lost (mean (SD))*  - Yoga: £374.2 (£1,142.5)  - Usual care: £1,201.8 (£2,550.0)  *Equipment purchase cost (mean (SD))*  (mattress, mattress topper, bed, chair, pillow, shoes)  - Yoga: £59.9 (£220.2)  - Usual care: £129.0 (£297.8) |
| Darlow 2019 | Total costs over 6 months  - FREE intervention: NZD2,387 (95%CI 1,334 to 3,433)  - Usual care: NZD3,069 (95%CI 1,891 to 4,247)  *Health system costs*  - FREE intervention: NZD665 (95%CI 330 to 999)  - Usual care: NZD997 (95%CI 642 to 3,424)  *Accident Compensation Corporation (ACC) costs*  - FREE intervention: NZD114 (95%CI -216 to 444)  - Usual care: NZD997 (95%CI 238 to 3,439) | NR |
| Geraghty 2024 | Total back pain-related costs  - SupportBack self-management: £182  - SupportBack self-management + telephone support: £280  Treatment costs/person  - SupportBack self-management: £16  - SupportBack self-management + telephone support: £61 | NR |
| Haake 2007 | Treatment costs  -Verum acupuncture: USD$721.26  - Standard therapy: USD$775.10 | NR |
| Herman 2017 | Treatment costs/person (mean)  - Cognitive-Behavioral Therapy (CBT): USD$150  - Mindfulness-Based Stress Reduction (MBSR): USD$150  - Usual care: USD$0  Total overall healthcare (payer) costs (mean (95%CI))  - CBT: USD$2,760 ($896 to $5,613)  - MBSR: USD$1,283 (−$263 to $3,011)  - Usual care: USD$2,265 ($778 to $5,542)  Total back-related healthcare (payer) costs, including cost of intervention (mean (95%CI))  - CBT: USD$1,683 ($436 to $4,380)  - MBSR: USD$572 ($66 to$2,250)  - Usual care: USD$699 ($4 to $3,763)  *All office-based and outpatient care (mean (95%CI))*  - CBT: USD$305 (-$31 to $911)  - MBSR: USD$-17 (-$208 to $303)  - Usual care: USD$219 (-$31 to $973)  *Emergency department (mean (95%CI))*  - CBT: USD$3 (-$42 to $100)  - MBSR: USD$12 ($0 to $36)  - Usual care: USD$-14 (-$61 to $0)  *Hospital inpatient (mean (95%CI))*  - CBT: USD$1,195 (95%CI $304, $3,231)  - MBSR: USD$407 (95%CI $81, $1,778)  - Usual care: USD$463 (95%CI $0, $2,693)  *Pharmacy (mean (95%CI))*  - CBT: USD$3 (-$18 to $35)  - MBSR: USD$-9 (-$28 to $11)  - Usual care: USD$10 (-$4 to $27)  *Imaging (mean (95%CI))*  - CBT: USD$26 (-$11 to $124)  - MBSR: USD$29 (-$11 to $97)  - Usual care: USD$22 (−$16 to $78)  Total societal costs*(mean (95%CI))  - MBSR: USD$6428 ($3,676 to $1,0262)  - CBT: USD$5580 ($3,465 to $8,343)  - Usual care: USD$6,304 ($4,193 to $9,805)  *Total Societal costs = Total overall healthcare costs + Patient copay amounts + Lost productivity from absenteeism + Lost productivity from presenteeism | *Lost productivity from absenteeism (mean (95%CI))*  - CBT: USD$1,004 ($411 to $2,008)  - MBSR: USD$1,092 ($535 to $1,945)  - Usual care: USD$803 ($377 to $1,528)  *Lost productivity from presenteeism (mean (95%CI))*  - CBT: USD$2,603 ( $3,96 1to $3,961)  - MBSR: USD$3,153 ($4,441 to $4,441)  - Usual care: USD$3,254 ($4,768 to $4,768) |
| Hochheim 2021 | Minor impairment  Total costs  - Multidisciplinary biopsychosocial rehabilitation (MBR): €15,749  - Usual care: €11,761  *Back pain specific costs*  - MBR: €2,143  - Usual care: €1956  *Inpatient costs- back pain*  - MBR: €186  - Usual care: €190  *Outpatient cost- back pain*  - MBR: €1,957  - Usual care: €1,766  Major impairment  Total costs  - MBR: €13,805  - Usual care: €18,543  *Back pain specific costs*  - MBR: €2,296  - Usual care: €3,533  *Inpatient costs- back pain*  - MBR: €336  - Usual care: €926  *Outpatient cost- back pain*  - MBR: €1,960  - Usual care: €2,606 | NR |
| Herman 2026 | Total healthcare sector costs (mean (95%CI))  -Standard Acupuncture (SA): USD $881 ($39 to $2,350)  -Enhanced Acupuncture (EA): USD −$369 (−$2,374 to $951)  -Usual care: USD $122 (−$819 to $1,529)  Total patient copayments and coinsurance (mean (95%CI))  - SA: USD−$113 (−$173 to −$65)  - EA: USD−$101 (−$136 to $58)  -Usual care: USD−$31 (−$70 to $21)  Total Medicare costs (mean (95%CI))  - SA: USD$993 ($156 to $2,465)  - EA: USD−$268 (−$2,271 to $1,034)  -Usual care: USD$153 (−$771 to $1,560)  Intervention (mean (95%CI))  -SA: USD $985 ($939 to $1,030)  - EA: USD $1,422 ($1,354 to $1,482)  -Usual care: USD $0  Hospital inpatient (mean (95%CI))  - SA: USD$474 (−$330 to $1,880)  - EA: USD−$1,127 (−$3,056 to $162)  -Usual care: USD$249 (−$647 to $1,610)  Hospital ambulatory (mean (95%CI))  - SA: USD−$116 (−$279 to −$36)  - EA: USD−$85 (−$163 to $9)  -Usual care: USD$27 (−$45 to $113)  Outpatient clinic visits (mean (95%CI))  - SA: USD−$159 (−$253 to −$66)  - EA: USD−$179 (−$269 to −$63)  -Usual care: USD−$111 (−$173 to −$50)  Email, telephone and video visits (mean (95%CI))  - SA: USD−$57 (−$83 to −$35)  - EA: USD−$67 (−$87 to −$47)  -Usual care: USD−$38 (−$60 to –$17)  Pharmacy fills/refills (mean (95%CI))  - SA: −$31 (−$162 to $81)  - EA: −$165 (−$394 to −$23)  -Usual care: $38 (−$331 to $216)  Other costs (mean (95%CI))  - SA: USD $17 (−$30 to −$130)  - EA: USD $4 (−$11 to −$42)  -Usual care: USD−$10 (−$246 to $71) |  |
| Hollinghurst 2008 | Cost to NHS (mean (95%CI))  - Massage: £158.22 (80.39 to 236.05)  - Exercise: £44.07 (−13.04 to 101.17) | NR |
| Hurwitz 2002 | Treatment cost  - Physiotherapy: USD$406.02  - Spinal manipulation: USD$199.32 | NR |
| Johnson 2007 | Treatment cost  - CBT: £27  - Usual care: £0 | NR |
| Kent 2023 | All costs (mean (SD))  - Cognitive Functional Therapy (CFT): AUD$13,433 (20,665)  - CFT + biofeedback: AUD$11,878 (16,303)  - Usual care: AUD$17,401 (23,555)  Total health costs (mean (SD))  - CFT: AUD$6,638 (9,937)  - CFT + biofeedback: AUD$6,385 (11,041)  - Usual care: AUD$6,323 (11,428)  *Health professionals* (mean (SD))  - CFT: AUD$2,480 (2,867)  - CFT + biofeedback: AUD$2,667 (3,514)  - Usual care: AUD$2,963 (5,420)  *Health professionals* (mean (SD))  - CFT: AUD$772 (1,311)  - CFT + biofeedback: AUD$542 (674)  - Usual care: AUD$718 (977)  *Hospital* (mean (SD))  - CFT: AUD$2,466 (7,547)  - CFT + biofeedback: AUD$1,888 (7,379)  - Usual care: AUD$2,360 (8,296)  *Private travel* (mean (SD))  - CFT: AUD$69 (116)  - CFT + biofeedback: AUD$55 (83)  - Usual care: AUD$70 (136)  *Ambulance* (mean (SD))  - CFT: AUD$65 (479)  - CFT + biofeedback: AUD$18 (168)  - Usual care: AUD$93 (401) | Total productivity losses (mean (SD))  - CFT: AUD$7,267(14,698)  - CFT + biofeedback: AUD$6,592 (11,471)  - Usual care: AUD$11,323 (18,517)  *Presenteeism (mean (SD))*  - CFT: AUD$4,404 (8,253)  - CFT + biofeedback: AUD$4,841 (9,130)  - Usual care: AUD$8,451 (14,757)  *Absenteeism (mean (SD))*  - CFT: AUD$2,863 (9,728)  - CFT + biofeedback: AUD$1,750 (5,016)  - Usual care: AUD$2,873 (7,369) |
| Kim 2010 | Total cost  -Acupuncture+usual care: KRW969,471  -Usual care: KRW746,918  Direct medical cost  -Acupuncture+usual care: KRW433,432  -Usual care: KRW275,740  Direct non-medical cost  -Acupuncture+usual care: KRW296,897  -Usual care: KRW232,036 | Indirect non-medical cost  -Acupuncture+usual care: KRW239,142  -Usual care:KRW239,142 |
| Kongstad 2024 | Total costs (mean (SD))  Usual care: €2670 (5549)  SelfBack: €3762 (7887)  Total healthcare costs (mean (SD))  Usual care: €661.2 (892.0)  SelfBack: €878.8 (1137.4)  *Intervention costs (mean)*  Usual care: €0  SelfBack: €89 | Productivity loss (mean (SD))  Usual care: €1495 (4972)  SelfBack: €2377 (7596) |
| Lamb 2010 | Total healthcare costs (mean (95%CI))  - Cognitive behavioural programme + active management: £421.52 (378.11 to 468.70)  - Active management: £224.65 (176.99 to 275.66)  Overall mean cost of private health care and related costs (mean (95%CI))  - Cognitive behavioural programme + active management: £400.55 (292.16 to 541.27)  - Active management: £270.65 (205.87 to 350.63)  Total cost initial intervention (mean)  - Cognitive behavioural programme + active management: £203.99  - Active management: £17.42 | NR |
| Lambeek 2010 | Total costs (mean (SD))  - Integrated care: £13,165 (13,600)  - Usual care: £18,475 (13,616)  Total direct costs (mean (SD))  - Integrated care: £1,479 (1,133)  - Usual care: £1,262 (1,094)  *Primary care costs* (mean (SD))  - Integrated care: £1,251 (700)  - Usual care: £857 (758)  *Secondary care costs* (mean (SD))  - Integrated care: £124 (416)  - Usual care: £247 (425)  *Direct non-healthcare costs* (mean (SD))  - Integrated care: £104 (225)  - Usual care: £159 (325) | Total indirect costs  - Integrated care: £11,686 (12,553)  - Usual care: £17,213 (13,416) |
| Ratcliffe 2006 | Total costs (mean (SD))  - Traditional Chinese acupuncture: £471.10 (341.61)  - Usual care: £332.24 (426.50) | NR |
| Skonnord 2022 | Health care costs (mean (95%CI)) day 28  - Acupunture: USD$99 (85 to 113)  - Usual care: USD$89 (77 to 102)  Health care costs (mean (95%CI)) day 365  - Acupunture: USD$648(266 to 1,029)  - Usual care: USD$645 (401 to 890) | Societal perspective (mean (95%CI)) day 28  - Acupunture: USD$1,904 (1,126 to 2,683)  - Usual care: USD$2,495 (1625 to -3,365)  Health care costs (mean (95%CI)) day 365  - Acupunture: USD$5,869 (2,639 to 9,100)  - Usual care: USD$10,343 (3,403 to -17,283) |
| Suman 2019 | Total societal costs (including indirect) (mean (SD))  - Multifaceted eHealth strategy: Mean €8,444 (€820)  - Digital education (letter): Mean €8,979 (€619)  - Cost difference (95% CI): €-535 (€-2230 to €1172)  *Primary care (mean (SD))*  - Multifaceted eHealth strategy: Mean €340 (€26)  - Digital education (letter): Mean €405 (€26)  - Cost difference (95% CI): €-65 (€-134 to €-2)  *Secondary care (mean (SD))*  - Multifaceted eHealth strategy: Mean €478 (€228)  - Digital education (letter): Mean €229 (€42)  - Cost difference (95% CI): €249 (€58 to €515)  *Alternative care (mean (SD))*  - Multifaceted eHealth strategy: Mean €742 (€218)  - Digital education (letter): Mean €322 (€55)  - Cost difference (95% CI): €421 (€182 to €722)  *Medication (mean (SD))*  - Multifaceted eHealth strategy: Mean €29 (€7)  - Digital education (letter): Mean €44 (€9)  - Cost difference (95% CI): €-15 (€-45 to €-0.70)  *Intervention (mean (SD))*  - Multifaceted eHealth strategy: €70  - Digital education (letter): €0 (€0)  - Cost difference: €70 (N/A) | *Absenteeism*  - Multifaceted eHealth strategy: Mean €1,034 (€242)  - Digital education (letter): Mean €1,547 (€235)  - Cost difference (95% CI): €-513 (€-941 to €-77)  *Presenteeism*  - Multifaceted eHealth strategy: Mean €5,735 (€681)  - Digital education (letter): Mean €6,342 (€537)  - Cost difference (95% CI): €-607 (€-2076 to €-831)  *Unpaid Productivity*  - Multifaceted eHealth strategy: Mean €4,000 (€887)  - Digital education (letter): Mean €5,047 (€616)  - Cost difference (95% CI): €-1047 (€-1954 to €-203) |
| UK BEAM | Total cost (mean (SD)); - Best care in general practice: £346 (£602); - Best care + exercise: £486 (£907); - Best care + manipulation: £541 (£768); - Best care + manipulation + exercise: £471 (£490); *Spinal manipulation within trial (mean (SD))*; - Best care + manipulation: £147 (£53); - Best care + manipulation + exercise: £115 (£53); *Exercise class within trial (mean (SD))*; - Best care + exercise: £41 (£22); - Best care + manipulation + exercise: £37 (£22); *Hospital inpatient stay (mean (SD))*; *NHS routine admission*; - Best care in general practice: £28 (£148); - Best care + exercise: £31 (£197); - Best care + manipulation: £17 (£91); - Best care + manipulation + exercise: £19 (£114); *NHS emergency admission*; - Best care in general practice: £11 (£86); - Best care + exercise: £17 (£112); - Best care + manipulation: £20 (£128); - Best care + manipulation + exercise: £14 (£106); *Private hospital admission*; - Best care in general practice: £6 (£66); - Best care + exercise: £5 (£85); - Best care + manipulation: £5 (£71); - Best care + manipulation + exercise: £7 (£86); *Outpatient attendance (mean (SD))*; *NHS specialist*; - Best care in general practice: £63 (£186); - Best care + exercise: £63 (£234); - Best care + manipulation: £51 (£150); - Best care + manipulation + exercise: £34 (£121); *Private specialist*; - Best care in general practice: £14 (£85); - Best care + exercise: £42 (£470); - Best care + manipulation: £16 (£110); - Best care + manipulation + exercise: £13 (£110); *NHS physiotherapist*; - Best care in general practice: £22 (£103); - Best care + exercise: £27 (£195); - Best care + manipulation: £27 (£165); - Best care + manipulation + exercise: £11 (£60); *Private physical therapist**; - Best care in general practice: £56 (£280); - Best care + exercise: £44 (£335); - Best care + manipulation: £103 (£428); - Best care + manipulation + exercise: £49 (£216); *Other NHS*; - Best care in general practice: £20 (£146); - Best care + exercise: £11 (£62); - Best care + manipulation: £14 (£90); - Best care + manipulation + exercise: £12 (£69); *Other private*; - Best care in general practice: £23 (£145); - Best care + exercise: £28 (£195); - Best care + manipulation: £23 (£196); - Best care + manipulation + exercise: £13 (£79); *General practice consultation (mean (SD))*; *General practitioner*; - Best care in general practice: £88 (£139); - Best care + exercise: £63 (£129); - Best care + manipulation: £94 (£146); - Best care + manipulation + exercise: £73 (£118); *Practice nurse*; - Best care in general practice: £4 (£18); - Best care + exercise: £7 (£48); - Best care + manipulation: £9 (£44); - Best care + manipulation + exercise: £5 (£33); *Physiotherapist*; - Best care in general practice: £1 (£4); - Best care + exercise: £102 (£125); - Best care + manipulation: £4 (£28); - Best care + manipulation + exercise: £57 (£63); *Other*; - Best care in general practice: £11 (£55); - Best care + exercise: £5 (£30); - Best care + manipulation: £11 (£51); - Best care + manipulation + exercise: £10 (£44); *Including spinal manipulation or exercise class outside UK BEAM | NR |
| Verhaeghe 2018 | Treatment costs  - Osteopathy: €385.1  - Usual care: €501.8 | NR |
| Von Korff 2005 | Treatment costs  -Multidisciplinary intervention: USD$259.68  -Usual care: USD$0 | NR |
| Williams 2019 | Total costs including absenteeism (mean (SD))  - Coaching intervention: AUD$1,272 (135)  - Usual care: AUD$1,886 (683)  *Intervention costs (mean (SD))*  - Coaching intervention: AUD$8 (68)  - Usual care: AUD$0  *Healthcare costs (mean (SD))*  - Coaching intervention: AUD$355 (94)  - Usual care: AUD$648 (175)  *Medication costs (mean (SD))*  - Coaching intervention: AUD$119 (12)  - Usual care: AUD$149 (14) | Absenteeism costs (mean (SD))  - Coaching intervention: AUD$89 (68) - Usual care: AUD$1,089 (652) |

Currencies: AUD = Australian dollar; CAD = Canadian dollar; CHF = Swiss franc; NIS = New Israeli shekel; NOK = Norwegian Kroner; SGD = Singapore dollar; TRY = Turkish Liras; USD = United States of America dollar; £ = Pounds Stirling; € = Euro.
