# Supplementary material for: Cost-effectiveness of recommended interventions for managing low back pain: A systematic review
Source: PLoS Med. 2026 Oct 27;23(10):e1005273. doi: 10.1371/journal.pmed.1005273 (PMC13712551; doi:10.1371/journal.pmed.1005273)
Supplement: Supplementary file 6 [file pmed.1005273.s006.docx]

**Supplementary material 6: studies excluded from this report and reason for exclusion.**

| Study | Treatments investigated | Comparator | Reason for exclusion |
| --- | --- | --- | --- |
| Aboagye 2015 | Yoga or exercise therapy | Self-care advice | Comparison of two active interventions |
| Ademi 2016 | Primarily surgical treatment (PST) | Primarily conservative treatment (PCT) (physiotherapy, epidural injection, medication) | Sciatica |
| Bachmann 2009 | Function-centred treatment (FCT) (functional restoration programme of exercise) | Pain-centred treatment (PCT) (physical therapies, back school) | Comparison of two active interventions |
| Bello 2015 | Conventional exercise therapy (CET) | Behavioural graded activity (BGA) | Comparison of two active interventions |
| Borg 2021 | X-stop device | Laminectomy | Sciatica |
| Brown 1992 | Back school | No back school | Not RCT nor modelling study |
| Çakmak Başer 2020 | Inpatient physical therapy (electrotherapy, heat, exercise) or outpatient physical therapy | Back school | Not RCT nor modelling study |
| Canaway 2018 | Behaviour change plus physiotherapy | Usual care (physiotherapy) | Not RCT nor modelling study |
| Cherkin 1998 | Physical therapy (McKenzie), chiropractic manipulation | Educational booklet | Comparison of two active interventions |
| Choudhry 2022 | Multidisciplinary intervention (physical therapy, health coach counselling, and consultation from a specialist in pain medicine or rehabilitation) | Individualized postural therapy | Comparison of two active interventions |
| Cooley 2021 | Osteopathic manipulative treatment (OMT) | Usual care | Not RCT nor modelling study |
| Critchley 2007 | Physiotherapy (spinal stabilization classes, or physiotherapist-led pain management [structured back pain education with group strengthening, stretching, and graded exercises]) | Usual care (physiotherapy) | Comparison of two active interventions |
| Donelson 2019 | Quality-assured mechanical assessment (MC) | Usual community care (CC) | Not RCT not modelling study |
| Eisenberg 2007 | Usual care plus patient's choice of acupuncture or chiropractic or massage therapy | Usual care | Comparison of two active interventions |
| Fatoye 2020 | Telerehabilitation-based McKenzie therapy (TBMT) | Clinic-based McKenzie Therapy (CBMT) | Comparison of two active interventions |
| Fritz 2003 | Classification-Based Physical Therapy (CBPT) | Guideline-Based Therapy (GBT) | Comparison of two active interventions |
| Goossens 2015 | Cognitive behavioral treatment (Exposure in vivo (EXP) treatment) | Graded activity (GA) | Comparison of two active interventions |
| Haufe 2017 | Low-dose, non-supervised exercise | No intervention (wait-list control) | Does not report on primary outcome |
| Hemmila 2002 | Physiotherapy [individual thermal, electric, and manual treatments plus auto stretching exercises] or bone setting [traditional soft tissue, spinal, and joint manipulations] | Exercise [taught to interrupt static postures when not otherwise moving] | Comparison of two active interventions |
| Henchoz 2010 | Functional multidisciplinary rehabilitation plus exercise programme | Functional multidisciplinary rehabilitation plus usual care | Comparison of two active interventions |
| Hlobil 2007 | Graded activity (GA) + physical exercise session with a cognitive behavioral approach | Usual care | Does not report on primary outcome |
| Hurley 2015 | Exercise (group exercise class (EC) or supervised walking program (WP)) | Physiotherapy | Comparison of two active interventions |
| Karjalainen 2004 | Physician prescribed exercises (“mini-intervention”) or work-site visit | Usual care | Does not report on primary outcome |
| Kominski 2005 | Chiropractic care with and without Physical Modalities (DCPt) or medical care + physical modalities | Medical care | Does not report on primary outcome |
| Kool 2007 | Function-Centered Treatment (FCT) rehabilitation [strength, endurance, and cardiovascular training] | Pain-Centered Treatment (PCT) [mobilization, stretching, strength training, mini back school] | Comparison of two active interventions |
| Koppenaal 2023 | Blended Physiotherapy [e-physiotherapy] | Face-to-Face Physiotherapy | Comparison of two active interventions |
| Lee 2018 | Dorsal root ganglion block plus pulsed radiofrequency | Radiofrequency pulse therapy only | Comparison of two active interventions |
| Lewis 2005 | Individual exercise | Exercise for group | Comparison of two active interventions |
| Lewkowicz 2023 | Digital therapeutic care [unsupervised app-based treatments plus education] | Usual care | Not RCT nor modelling study |
| Luijsterburg 2007 | Physical therapy + GP usual care | Usual care (GP) | Sciatica |
| Malmivaara 1995 | Rest or exercise | Usual care | Does not report on primary outcome |
| Miyamoto 2018 | Pilates | Booklet group | Comparison of two active interventions |
| Moffett 1999 | Exercise | Usual care | Does not report on primary outcome |
| MoldeHagen 2003 | Advice | Usual care | Does not report on primary outcome |
| Nagel 2009 | Multimodal therapy program [physiotherapy, exercise, behavioral therapy, relaxation] | No intervention (pre-post design) | Not RCT nor modelling study |
| Niemisto 2005 | Combined treatment [manipulative treatment, stabilizing exercises, and physician consultation} | Physician consultation alone | Comparison of two active interventions |
| Nykänen 2004 | Group rehabilitation | Individually dosed rehabilitation | Not RCT/modelling study |
| Pennington 2020 | Epidural steroid injections (ESIs) | Medical management alone (without ESIs) | Sciatica |
| Pinnington 2004 | Physiotherapy [prompt access to] | No intervention (cohort design) | Not RCT/modelling study |
| Price 2005 | Epidural steroid plus local anaesthetic | Placebo (saline) | Sciatica |
| Rhon 2018 | Early physical therapy (PT) | Usual care | Does not report on primary outcome |
| Rivero-Arias 2006 | Physiotherapy with advice | Usual care (advice from physiotherapy) | Comparison of two active interventions |
| Rogerson 2010 | Interdisciplinary early intervention [cognitive-behavioral and physical therapy or cognitive-behavioral therapy] | Usual care | Does not report on primary outcome |
| Ronzi 2017 | Functional restoration program (FRP) [physiotherapist supervised exercise], or Ambulatory Individual Physiotherapy (AIP) [individualised home exercises] or combined strategy | Combined strategy (FRP + AIP) | Comparison of two active interventions |
| Schultz 2013 | Flexible early intervention [individual need-driven early intervention with the same modalities as the fixed approach] | Fixed early intervention [protocol-driven, interdisciplinary, multimodal, clinical, occupational, and case management-based] | Comparison of two active interventions |
| Schweikert 2006 | Cognitive-Behavioral Therapy (CBT) + rehabilitation + usual care | Rehabilitation + usual care | Comparison of two active interventions |
| Skouen 2002 | Light or extensive multidisciplinary treatment | Usual care | Does not report on primary outcome |
| Smeets 2009 | Active Physical Treatment (APT) and Graded Activity with Problem Solving Training (GAP) as single-treatment modalities | Combined strategy (APT + GAP) | Comparison of two active interventions comparison |
| Spijker-Huiges 2015 | Corticosteroid injection | Usual care | Sciatica |
| Steenstra 2006 | Workplace intervention or clinical intervention | Usual Care | Does not report on primary outcome |
| Strong 2006 | Lay delivered self-care intervention or psychologist delivered self-care intervention | Usual Care | Does not report on primary outcome |
| Teng 2019 | Hydrotherapy | Land-based therapy | Comparison of two active interventions |
| Torstensen 1998 | Medical Exercise Therapy (MET) or Conventional Physiotherapy (CP) | Self Exercise | Comparison of two active interventions |
| Vaegter 2020 | Cognitive Functional Therapy (CFT) | Multidisciplinary Pain Management (MPM) | Not RCT nor modelling study |
| Van Der Roer 2008 | Intensive physiotherapy protocol | Usual care (physiotherapy) | Comparison of two active interventions |
| Van Baal 2020 | Specific sensorimotor treatment (SMT) | General Exercise (GE) | Comparison of two active interventions |
| Vavrek 2014 | Spinal manipulation (SMT) | Light massage | Not RCT nor modelling study |
| Wayne 2019 | Integrative medicine approach [at Osher Clinical Center (OCC) tertiary hospital] | Usual Care | Not RCT nor modelling study |
| Whitehurst 2007 | Brief Pain management program (BPM) | Physical therapy | Comparison of two active interventions |
| Wright 2005 | Advice & education [back advice booklet and one session of advice] plus usual care from GP | Advice & education [back advice booklet and one session of advice] | Comparison of two active interventions |

Abbreviations: LBP = Low Back Pain; CLBP = Chronic LBP; NSCLBP = Non-Specific CLBP; CHF = Swiss Franc; QALY = Quality-Adjusted Life Year.
